# Which Socio-economic Conditions Drive the Selection of Agroforestry at the Forest Frontier?

**DOI:** 10.1007/s00267-021-01439-0

**Published:** 2021-02-12

**Authors:** Elizabeth Gosling, Thomas Knoke, Esther Reith, Alyna Reyes Cáceres, Carola Paul

**Affiliations:** 1 Institute of Forest Management, TUM School of Life Sciences Weihenstephan, Technische Universität München, Hans-Carl-von-Carlowitz-Platz 2, 85354 Freising, Germany; 2 Department of Forest Economics and Sustainable Land Use Planning, University of Göttingen, Büsgenweg 1, 37077 Göttingen, Germany

**Keywords:** Alley cropping, Goal programming, Panama, Robust optimisation, Scenario analysis, Silvopasture

## Abstract

Models are essential to assess the socio-economic credentials of new agroforestry systems. In this study, we showcase robust optimisation as a tool to evaluate agroforestry’s potential to meet farmers’ multiple goals. Our modelling approach has three parts. First, we use a discrete land-use model to evaluate two agroforestry systems (alley cropping and silvopasture) and conventional land uses against five socio-economic objectives, focusing on the forest frontier in eastern Panama. Next, we couple the land-use model with robust optimisation, to determine the mix of land uses (farm portfolio) that minimises trade-offs between the five objectives. Here we consider uncertainty to simulate the land-use decisions of a risk-averse farmer. Finally, we assess how the type and amount of agroforestry included in the optimal land-use portfolio changes under different environmental, socio-economic and political scenarios, to explore the conditions that may make agroforestry more attractive for farmers. We identify silvopasture as a promising land use for meeting farmers’ goals, especially for farms with less productive soils. The additional labour demand compared to conventional pasture, however, may prove an important barrier to adoption for farms facing acute labour shortages. The selection of agroforestry responded strongly to changes in investment costs and timber prices, suggesting that cost-sharing arrangements and tax incentives could be effective strategies to enhance adoption. We found alley cropping to be less compatible with farmers’ risk aversion, but this agroforestry system may still be a desirable complement to the land-use portfolio, especially for farmers who are more profit-oriented and tolerant of risk.

## Introduction

Agroforestry is a multifunctional form of agriculture that combines trees and crops and/or livestock on the same parcel of land. These systems are often advocated as a sustainable land-use strategy to reduce poverty, mitigate climate change and improve food security in tropical regions (Leakey 2020; Montagnini and Metzel 2018; Waldron et al. 2016). For example, in the Central American Republic of Panama, the government promotes agroforestry within its private–public initiative to restore 1 million hectares of forest land (“Alianza por el Millón”; Garcia et al. 2016; MiAmbiente 2019). This has included enacting a legal framework for tax exemptions and subsidies for agroforestry systems (Law No. 69 of October 30, 2017). However, the uneven and relatively slow uptake of agroforestry in Central and Latin America (Dagang and Nair 2003; Frey et al. 2012a; Somarriba et al. 2012) suggests that not all farmers deem these systems to be a desirable land-use option (Do et al. 2020). While the ecological advantages of agroforestry have been widely documented (e.g., Jose 2009), the socio-economic disadvantages that may constitute barriers to adoption have received less attention in the literature (Liu et al. 2019; Montambault and Alavalapati 2005). More research to better understand the socio-economic aspects of agroforestry is therefore needed, to help identify conditions that may make agroforestry more attractive for farmers.

Given the cost and risks associated with field experiments, models are an important tool to assess the socio-economic potential of different agroforestry systems, to pre-select the most promising systems for on-farm trials (Bertomeu and Giménez 2006; Kaim et al. 2018; Le Gal et al. 2011). Within this context, goal programming has two advantages for evaluating agroforestry. First, as a multi-criteria decision analysis (MCDA) method, goal programming can consider multiple objectives and hence account for the diverse, and potentially conflicting, goals that drive farmers’ decision-making (Janssen and van Ittersum 2007; Kaim et al. 2018; van Zonneveld et al. 2020). Second, as a continuous (rather than discrete) MCDA technique, goal programming can solve land allocation problems to simulate decision-making at the farm (rather than plot) level. For example, goal programming can be used to determine the optimal mix of land uses to achieve a set of objectives (Janssen and van Ittersum 2007; Uhde et al. 2015). This farm-level modelling accounts for land-use diversification, a common strategy among smallholders to meet different household needs (Knoke et al. 2017; Pannell et al. 2014) and reduce risk (Baumgärtner and Quaas 2010; Di Falco and Perrings 2005).

Goal programming can therefore complement previous modelling approaches that have evaluated agroforestry against socio-economic and ecological objectives at the plot level, but ignored the effects of land-use diversification on farmers’ decision-making (e.g., Palma et al. 2007; Rahman et al. 2017; Santos Martin and van Noordwijk 2011). Conversely, by considering multiple objectives, goal programming can enrich previous economic analyses that account for diversified land-use portfolios (farm-level modelling), but only assess agroforestry against a single criterion of profit maximisation and/or risk reduction. This includes studies based on Markowitz’s (1952) Modern Portfolio Theory (e.g., Bertomeu and Giménez 2006; Blandon 2005; Ochoa et al. 2016; Paul et al. 2017).

While goal programming has recently emerged as a tool to solve allocation problems in forestry (e.g., Aldea et al. 2014; Diaz-Balteiro and Romero 2008; Messerer et al. 2017) and agriculture (e.g., Ballarin et al. 2011; Biswas and Pal 2005; Knoke et al. 2015), applications to evaluate agroforestry are rare (García-de Ceca and Gebremedhin 1991; Mendoza et al. 1987). Recently, Gosling et al. (2020a, b) and Reith et al. (2020) used a variant of goal programming to investigate the role of agroforestry in optimised land-use portfolios that reduce trade-offs between different farm- and landscape-level objectives at the forest frontier in eastern Panama. These recent studies, however, relied solely on perception data from local farmers and relevant experts. Such data sets help us to understand the extent to which farmers perceive different agroforestry systems to be compatible with their objectives, but are less helpful for understanding the factors that could promote greater uptake of agroforestry. This is because it is unknown how farmer perceptions would change in response to market developments, policy interventions or changing environmental and household conditions. Perception data may also tend to reflect what farmers deem desirable, rather than what is actually feasible given their hard economic constraints (Gosling et al. 2020b). Moreover, farmers may find it difficult to appraise agroforestry systems with which they are not yet familiar given the complexity and long planning horizons of these systems (Do et al. 2020; Laroche et al. 2018).

To address these shortcomings, the current study couples goal programming with more detailed socio-economic coefficients to explore the conditions that may favour the adoption of agroforestry at the tropical forest frontier. Such socio-economic coefficients, which we derived from land-use models, may provide a more neutral basis to simulate decision-making, one which can more easily capture farmers’ hard economic constraints as well as changing environmental or market conditions (such as poorer soils or rising timber prices). Our guiding research question is: Which environmental and socio-economic conditions drive the selection of agroforestry in a diversified farm portfolio that reduces trade-offs between multiple objectives under uncertainty? Exploring this question may reveal potential leverage points for increasing agroforestry adoption among different types of farmers, to inform the design of incentive schemes and help target extension programs.

## Methods

**Fig. 1:**
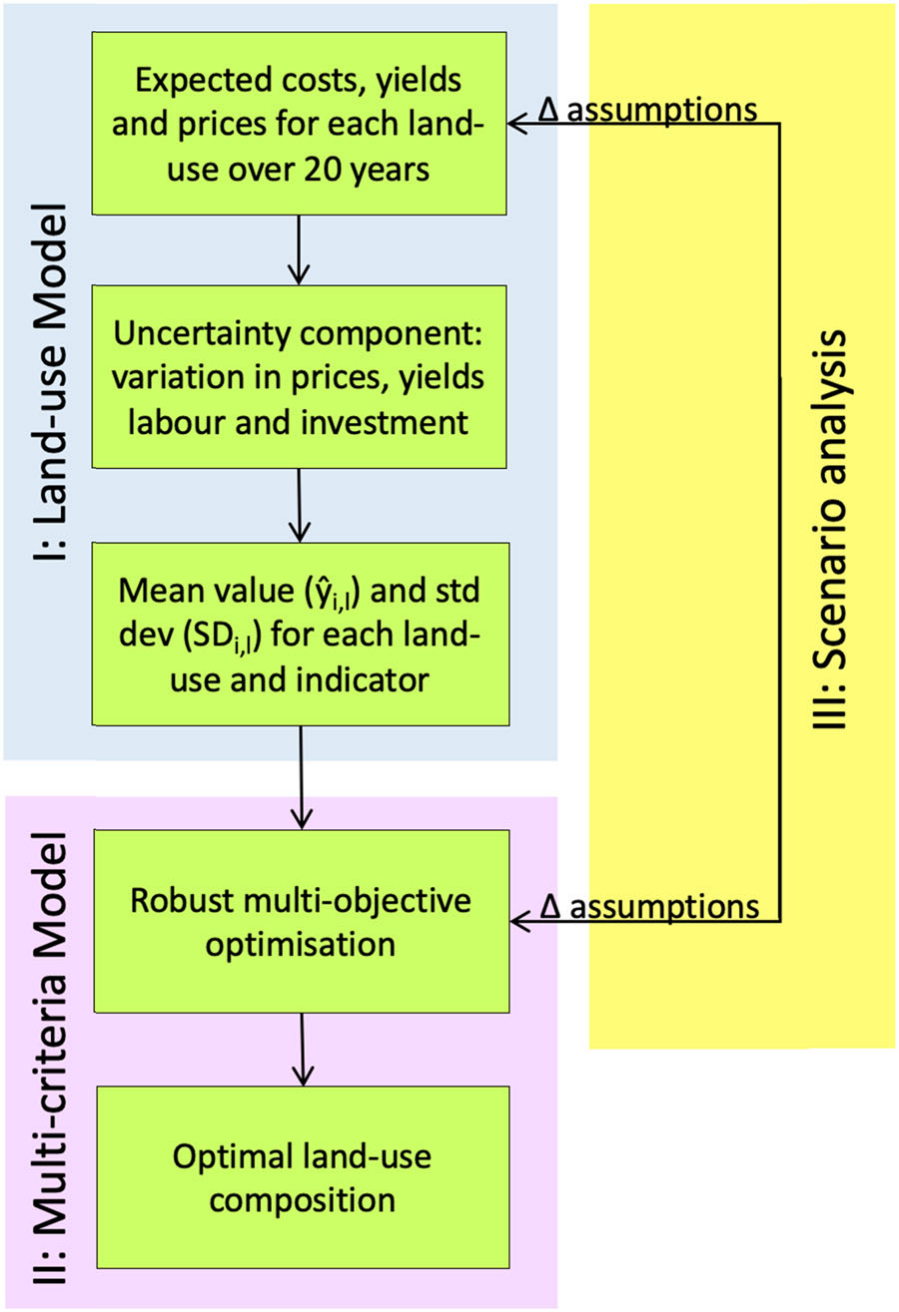
The three components of the multi-criteria analysis

We evaluate the potential of agroforestry to meet farmers’ socio-economic goals in three steps (shown by the blue, mauve and yellow sections of Fig. 1). First we develop a discrete land-use model to quantify the performance of seven mutually exclusive land uses (including two agroforestry options) against five pre-defined, socio-economic indicators. Our land-use model integrates national data from Panama with measured and modelled data from the study area. It combines deterministic capital budgeting with Monte Carlo simulations to account for variability in inputs, outputs and prices. Using the land-use model, we generate predicted (mean) values, $\hat y_{i,l}$, and associated standard deviation SD_*i,l*_, for each land use, *l*, for each indicator, *i*.

These values form the input data for the second stage of modelling: robust multi-criteria optimisation, a variant of goal programming. The five pre-defined indicators serve as farmers’ objectives and represent our decision criteria in the multi-criteria (optimisation) model. The area shares of each land use within a hypothetical farm are the decision variables. The multi-criteria model selects the theoretically optimal mix of land uses (which we refer to as a land-use portfolio) for balancing the achievement of the five socio-economic objectives when accounting for uncertainty. Our optimisation approach follows Gosling et al. (2020a, b), but is expanded to include more detailed socio-economic coefficients for a wider range of land-use alternatives.

We further extend the modelling approach through a scenario analysis in the third part of the study (Fig. 1); here we modify parameters within the land-use and multi-criteria models to simulate different household, environmental, market and political conditions. We analyse how the type and amount of agroforestry selected in the optimal portfolio changes under these different scenarios, to better understand the factors and conditions that may make agroforestry more (or less) attractive for different farmers.

### Study Area, Selected Land Uses and Indicators

We demonstrate our modelling approach for Tortí, a farming region in eastern Panama, near the border of the Panamá and Darién provinces. Tortí lies in the humid tropical zone, receiving 1900 mm rainfall per year, concentrated between April and December (ETESA 2018). Our study area covers around 9100 ha. The terrain is mostly flat at around 100 m above sea level; hills to the southeast rise to 400 m in elevation (ANAM 2011). Soils originate from sedimentary rock, including tertiary limestone, arenite and lutite, and are classified as vertisols, cambisols and nitisols (ANAM 2011; Gardi et al. 2015; Paul 2014).

The region is one of the last forest frontiers in Central America to undergo intense colonisation (Peterson St-Laurent et al. 2013). Colonists from Panama’s western provinces began to settle the area in the 1970s, marking the start of widespread forest clearing (Paul 2014; Sloan 2008). Cattle grazing and agriculture now dominate the landscape; pasture and cropland comprise 60 and 26% of farmland in Tortí (Gosling et al. 2020a). Large-scale forest plantations of the exotic species teak (*Tectona grandis*) are also common in the study area, usually owned by foreign companies (Sloan 2008). The remaining natural forest cover comprises 14% of farmland (Gosling et al. 2020a).

Table 1 outlines the seven land uses investigated in this study. Following Odum’s (1969) classic paper (Corman et al. 2019), we classify these land uses into productive, compromise and protective landcover types. We investigate four productive land uses: pasture for cattle grazing, rice (*Oryza sativa*) and maize (*Zea mays*), which are the most commonly grown annual crops in the study area (Duarte 2018), and teak plantation.

**Table 1:** Description of the seven land uses, *l*, selected in this study

Classification	Name	Description	Sources
Productive	Rice Maize	Traditional non-mechanised and non-irrigated system, with the use of fertiliser and pesticides: crops planted and harvested once per year.	MIDA (2019a, b)
	Pasture	Cows graze on improved pasture (*Brachiaria* spp) with a stocking rate of 2 animals per hectare. *Ceba* (Spanish for mast) system, whereby young cows are bought, fattened on pastures and sold the following year.	Paul (2014) and Reyes Cáceres (2018)
	Teak plantation	Monoculture of teak (*Tectona grandis*) planted at density of 1110 trees per hectare. Trees undergo two thinnings with a final harvest after 20 years.	Paul et al. (2017)
Compromise	Alley cropping	Maize is grown between rows of teak trees, until canopy shading prevents crop cultivation. Teak is planted at a density of 555 trees per hectare, it undergoes two thinnings with a final harvest after 20 years.	Paul et al. (2017)
	Silvopasture	Same production system as conventional pasture, but pastures are planted with the native tree species Spanish cedar (*Cedrela odorata*) at a density of 200 trees per hectare. Trees are harvested for timber after 20 years.	Paul (2014) and Reyes Cáceres (2018)
Protective	Forest	Natural secondary forest of native species. No active management, cannot be used for commercial timber production.	INEC (2011)

Classification categories refer to the framework of Odum (1969)

As compromise land uses, we investigate two agroforestry systems: alley cropping and silvopasture. These systems represent novel land uses, because they are not yet widespread in the study area. Currently, the most common forms of agroforestry practiced in Tortí are home gardens, living fences and scattered trees in pastures (Gosling et al. 2020a; Schuchmann 2011). Our silvopasture system represents a more intensive system with 200 trees per hectare. We selected the native tree Spanish cedar (*Cedrela odorata*) for the silvopastoral system based on its potential to produce high-value timber and local farmers’ preference for this species (Reyes Cáceres 2018). The alley cropping system comprises rows of teak trees with maize cultivated in between. The species selection and layout are based on a local trial coupled with bio-economic modelling, which found this alley cropping system to be an economically competitive land use in the study area (Paul et al. 2015, 2017). Because canopy shading prevents maize production in the later part of the rotation, this tree–crop system can also be viewed as a taungya system (Fischer and Vasseur 2000; Paul et al. 2015).

Natural forest is a protective land use. It represents a landcover without active management, and therefore is not associated with any management costs or revenues. Forest can also be considered as long-term natural succession.

We aim to simulate the land-use decisions of a risk-averse farmer, who strives to reduce trade-offs between multiple farm-level objectives. We selected five hypothesised socio-economic objectives based on previous research in Panama and the tropics: (1) increasing long-term income, (2) maintaining frequent cash flows, (3) increasing food production, (4) reducing labour demand and (5) reducing investment costs. These objectives reflect factors likely to influence farmers’ land-use decisions, including their uptake of agroforestry (Connelly and Shapiro 2006; Fischer and Vasseur 2002; Holmes et al. 2017; Tschakert et al. 2007). We quantified the contribution of each land use for achieving each objective through the five indicators described in Table 2. Following Paul et al. (2017) and Pearce et al. (2003), we selected a 5% discount rate to calculate the net present value (NPV) and payback period of each land use.

**Table 2:** The five indicators, *i*, used to quantify the contribution of each land use for achieving the five pre-defined socio-economic objectives

Indicator	Unit	Direction	Rationale	Calculation
Net present value (NPV)	$/ha	More is better	Quantifies profitability for the objective of increasing long-term income. Profitability is an important characteristic influencing the adoption of land-use systems (Connelly and Shapiro 2006; Coomes et al. 2008).	Sum of all discounted net cash flows (NCF) over a 20-year period, using a 5% discount rate: $${\rm{NPV}}_l = \mathop {\sum}\nolimits_t^T {{\rm{NCF}}_{l,t} \times (1.05)^{ - t}}$$
Payback period	Years	Less is better	We use payback period, i.e. the time taken to earn back the initial investment, to account for cash flow and access to money (Coomes et al. 2008; Holmes et al. 2017). This indicator relates to the objective of maintaining frequent cash flows.	As per Knoke et al. (2014), we compute a discounted payback period, defined as the 1st year (within the 20-year rotation) that has a positive discounted cumulative cash flow, based on a 5% discount rate.
Food production	Mcal/ ha/yr	More is better	Smallholders’ land-use decisions may be constrained by the need to meet household food needs (Binh et al. 2008; Fischer and Vasseur 2002; Tschakert et al. 2007).	Mean annual energy production over a 20-year period: we convert crop and meat yields to dietary energy (Mcal per hectare) using the USDA (2019) food composition database and technical conversion factors for agricultural commodities (FAO 2019)—see Table S10.
Labour demand	Days/ ha/yr	Less is better	Labour availability can be a key constraint for land-use decisions of smallholder farmers (Pichón 1997; Tschakert et al. 2007; van Zonneveld et al. 2020).	The mean number of labour days required to implement and manage a given land use per year (averaged over a 20-year period).
Investment costs	$/ha	Less is better	Given a lack of capital among smallholder farmers, high investment costs pose a potential barrier to agroforestry adoption (Calle et al. 2009; Connelly and Shapiro 2006; Coomes et al. 2008).	Sum of all costs incurred in year 0 of the land-use model.

Direction refers to the desired state of an indicator, i.e., whether higher or lower values are preferable

### Land-use Model

To quantify the performance of each land use (Table 1) against each indicator (Table 2), we collated a data set outlining the expected costs, yields, producer prices and labour requirement of each land use for each year of a 20-year period. We captured variability in these inputs and outputs through Monte Carlo simulations, basing yield and price fluctuations on historical data series. The assumptions and input data of the land-use model draw on our experience from a local field trial (Paul 2014) and subsequent financial analysis (Paul et al. 2015) and bio-economic modelling (Paul et al. 2017) of tree–crop and conventional land-use systems in the study area.

#### Expected costs and revenues

The establishment costs for each land use (except native forest) include the costs of clearing secondary vegetation and weeds from fallow land. All labour costs are based on a daily wage of US$17.33, the current salary for agricultural workers in Panama (MIDA 2019a). Costs of purchasing land and taxes are excluded. All costs and revenues are presented on a per hectare basis and given in US$/ha, shortened to $/ha from here on.

The expected labour and input costs, yields and producer prices for agricultural crops were taken from technical notes from the Ministry of Agricultural Development of Panama (Ministerio de Desarrollo Agropecuario de Panamá, MIDA)—see Tables S1, S2 and S5 in the Supplementary material. These technical notes are compiled at the national level, but we selected the costs and yields for traditional (non-mechanised) planting techniques with some chemical inputs, which previous research identified as the common farming practice in Tortí (Gosling et al. 2020a; Paul et al. 2015; Schuchmann 2011). Costs for fencing and establishing pasture, as well as expected beef yields and prices, are based on national information from MIDA (2016) and adjusted to local conditions according to data from Paul et al. (2015) and experiences of key informants in the study area (see supplementary Tables S2 and S5).

Table 3 outlines the management regime for the three timber-based systems. Because Spanish cedar is susceptible to damage from the moth *Hypsipyla grandella*, which can reduce timber quality (Cordero and Boshier 2003), intensive pest management is carried out in the first 3 years to minimise damage. Following Paul (2014), cedar trees are then pruned annually in years 4–7. All management costs are detailed in Table S2. Timber prices for teak and cedar were obtained from the National Forest Office (ONF 2019) in Costa Rica—see Table S6.

**Table 3:** Thinning and pruning regimes for the three timber land-use systems (following Paul 2014 and Paul et al. 2017)

	Pure plantation	Alley cropping	Silvopasture
Species	*T. grandis*	*T. grandis* and *Z. mays*	*C. odorata*
Planting layout (tree spacing)	3 × 3 m	3 × 6 m	7 × 7 m
Initial tree density (stems/ha)	1110	555	200
Tree pruning (years after establishment)	1,2,4	1,2,3,5	4–7
Thinning	Year 4: 60% Year 10: 50%	Year 5: 50% Year 10: 50%	none
Final stem number (stems/ha)^a^	222	139	200

^a^Excluding tree mortality

Following Paul et al. (2015), we extrapolated the annual height and diameter growth (and thus net increment in standing timber volume) of teak and cedar in the pure plantation and agroforestry systems from initial growth data in the study area (Paul 2014). We assumed an annual tree mortality rate of 0.5% (Griess and Knoke 2011). To simulate shading in the alley cropping and silvopastoral systems, we extrapolated canopy development from the same initial growth data (for teak and cedar, respectively) using regression with diameter (dbh) as the predictor (Paul et al. 2015).

In the timber-based systems farmers clear all vegetation within a 1 m radius of each tree seedling, to reduce light and competition effects (Paul et al. 2015). This reduces the total area available for maize cultivation by 17% in the alley cropping system compared to the monoculture: we reduced the per hectare cultivation costs and expected yields of maize accordingly. Similarly, in the silvopasture system 5% less area is available for pasture, reducing the initial stocking rate to 1.9 cows per hectare.

We modelled the further reduction in maize yields due to canopy shading using the categories devised by Paul et al. (2015) that account for height and canopy development of teak trees (Table S4). Following this method, there was sufficient light for maize to be cultivated in the initial year of tree planting and the first 2 years thereafter (during which time we expect full yields). Canopy shading then prevents maize cultivation for the remainder of the rotation, except for in the years immediately following thinning (years 6 and 11 after tree establishment), when expected yields are reduced by a factor of 0.5. The alley cropping system accounts for economies of scope with reduced weeding costs for trees during maize cultivation. Furthermore, lower chemical inputs are required for maize in the alley cropping system compared to the monoculture, because maize is not cultivated every year (see Section 1.1 in the Supplementary material for details).

To account for the effect of shading on pasture productivity, we assume a 50% yield reduction of pasture underneath the tree canopy. This is likely to be a conservative assumption, because in the early years of the rotation when tree canopies are still sparse, low levels of shading may actually enhance pasture productivity (Andrade et al. 2008; Fassola et al. 2006) and potentially extend the growing season (Jose et al. 2017). We reduce the stocking rate, *S*_*t*_, in the silvopasture system in year *t* of the rotation linearly:

1 $$S_t = S_0 \times \frac{{A_{{\rm{sun}},t} + \left( {0.5 \times A_{{\rm{canopy}},t}} \right)}}{{A_0}}$$

where *A*_0_ is the initial area of pasture, and *A*_sun,*t*_ and *A*_canopy,*t*_ the area of pasture in full sunlight and under the cedar canopy at year *t* of the rotation. By year 20, 36% of the initial pasture area is under the canopy of the cedar trees, reducing the stocking rate to 1.55 cows per hectare (see Fig. S2).

#### Variability in price, yields, labour demand and investment costs

The expected costs and revenues outlined above form the deterministic part of the land-use model. However, we also integrate an uncertainty component to capture inter-annual fluctuations in yields and prices (to reflect variable environmental conditions and the volatility of agricultural and timber markets), as well as potential variation in labour demand and investment costs (to reflect variability in inputs). For each year, *t*, considered in the land-use model, we adjust the expected yields and prices by bootstrapping from historical yield and price data for Panama (data from years 1997 to 2016: see Tables S8 and S9 as well as Eqs. (S2) and (S3) in the Supplementary material for further details). We also assume a 10% coefficient of variation for the investment costs and average labour demand of each land use. Using a Monte Carlo simulation with 10,000 repetitions, we then generate a frequency distribution of values of each indicator, *i*, for each land use, *l*. From these frequency distributions we can derive the mean scores $\hat y_{i,l}$ and standard deviations, SD_*i,l*_, which form the input data for our multi-criteria optimisation model (Table 4).

**Table 4:** Mean (predicted) value $\hat y_{i,l}$ and standard deviation SD_*i,l*_ derived from the Monte Carlo simulations for each land use, *l*, for each indicator, *i*

	NPV ($/ha)	Payback period (years)	Food production (Mcal/ha/year)	Labour demand (days/ha/year)	Investment costs ($/ha)
Rice	8310 ± 1756	0 ± 0.4	6295 ± 143	32 ± 0.7	949 ± 95
Maize	8066 ± 2643	1 ± 1.6	9866 ± 417	22 ± 0.5	1073 ± 109
Pasture	3496 ± 522	5 ± 1.1	976 ± 3	8 ± 0.2	1433 ± 142
Teak plantation	5267 ± 2019	20 ± 0.0	0 ± 0	16 ± 0.6	2184 ± 218
Alley cropping	5690 ± 1792	8 ± 8.6	1551 ± 141	12 ± 0.4	1835 ± 185
Silvopasture	4914 ± 696	11 ± 2.8	814 ± 2	14 ± 0.4	1970 ± 196
Forest	0 ± 0	0 ± 0.0	0 ± 0	0 ± 0.0	0 ± 0

Data represent the socio-economic coefficients used in the baseline scenario of our optimisation

### Multi-criteria Optimisation Model

The multi-criteria optimisation model selects the mix of land uses (defined by their area share in a hypothetical farm portfolio) that minimises trade-offs between the five socio-economic objectives. Our optimisation approach, which is a variant of goal programming, was first developed by Knoke et al. (2015, 2016) for land allocation problems in tropical regions. The model is formulated as a min-max problem (Romero 2001). For each indicator, we set the best possible performance as our target level, and the model selects a land-use composition that minimises the worst shortfall between the target level and achieved level across all indicators. This results in a balanced solution where high levels of one indicator do not compensate for low levels of another (Romero 2001). A min-max formulation simulates “satisficing”—a mix between satisfying and optimising—behaviour, which can be a good match for farmer decision-making (Knoke et al. 2020b; Le Gal et al. 2011).

Uncertainty is an important influence on farmers’ decisions, especially as a driver of land-use diversification (Baumgärtner and Quaas 2010). Such uncertainty relates in part to our inability to know exactly how much a land use will contribute to a given objective, either now or in the future. We account for uncertainty through robust decision-making. When seeking the best solution, the optimisation model not only considers the predicted performance of each land use for achieving each objective ($\hat y_{i,l}$), but also potential fluctuations in this performance. The model then finds solutions that secure minimum levels of each objective, even in worst-case scenarios. However, we do not allocate probabilities to the predicted and worst-case scenarios. This form of non-stochastic, robust decision-making is often recommended when facing high levels of uncertainty (Walker et al. 2013).

The model computes potential fluctuations in land-use performance by adding or subtracting multiples, *m*, of the standard deviation, SD_*i,l*_ to or from the mean value of each land use, $\hat y_{i,l}$. For “less is better indicators”, we add a multiple of the standard deviation to the mean, while for “more is better” indicators, we subtract a multiple (see Eq. (S6)). In this way, we always compute an unfavourable deviation from the mean. The factor *m* controls the size of these unfavourable deviations and hence the level of uncertainty considered in the model. We carry out the optimisation for three different uncertainty levels: *m* = 0, which ignores uncertainty (the model considers mean scores only), reflecting the decision-making of a risk neutral farmer; *m* = 1.5 representing a moderate level of uncertainty, which could reflect the perspective of a moderately risk-averse farmer; and *m* = 3.0 reflecting a high level of uncertainty and the decision-making of a strongly risk-averse farmer.

The mathematical formulation of the optimisation model is outlined in Section 6 of the Supplementary material, but we also refer the reader to Gosling et al. (2020a) and Knoke et al. (2020a) for further details of the optimisation approach.

To check the plausibility of baseline model results, we compare the optimised portfolio to the current land-use composition of the study area, using the Bray–Curtis measure of dissimilarity. We computed the Bray–Curtis measure, BC_*o,c*_, based on the land-use area shares, *a*_*l*_, of the optimal (index *o*) and the current (index *c*) land-use portfolios (as recorded by Gosling et al. 2020a). BC_*o,c*_ values close to 0 indicate low dissimilarity and values close to 1 high dissimilarity:

2 $${\rm{BC}}_{o,c} = \frac{{\mathop {\sum }\nolimits_{l = 1}^7 \left| {a_{l,o} - a_{l,c}} \right|}}{2}$$

### Scenario Analysis

The optimal portfolio represents the land-use composition that best reduces trade-offs between the five socio-economic objectives, accounting for different levels of risk aversion. The data outlined in Table 4 represent the socio-economic coefficients used in the baseline scenario of our optimisation. In the third part of the study, we rerun the optimisation for a series of scenarios (outlined in Table 5) that reflect different household, environmental, market and political conditions. For all scenarios we follow the principle of ceteris paribus, changing one variable or element at a time, to test how this change influences the type and amount of agroforestry included in the optimal portfolio.

**Table 5:** Overview of the scenarios tested in the sensitivity analysis

Type	Scenario name	Description	Changes in socio-economic coefficients	Justification
Assumptions of multi-criteria model	Prioritising individual objectives	The five indicators are no longer weighted equally in the optimisation. Instead we test the impact of making one indicator twice as important as the others. Weighting method described in Section 7 of Supplementary material.	None: all values as per Table 4.	Simulates the decision-making of a farmer who has a clear preference for one objective, but still considers the other household goals in their decision-making. Investigates how prioritising individual objectives may promote or hinder agroforestry adoption.
	Investment constraints	Introduce a constraint to restrict the total investment costs (per hectare) of the optimal portfolio.	None: all values as per Table 4.	In the baseline scenario, the multi-criteria model balances reducing labour demand and investment costs with the other socio-economic objectives. Optimal portfolios may exceed the labour availability and investment capacity of individual farms. For these scenarios, we set a limit for labour demand and investment costs, which the optimal portfolio cannot exceed. This is intended to simulate hard economic constraints.
	Labour constraints	Introduce a constraint to restrict the total labour demand (per hectare) of the optimal portfolio.	None: all values as per Table 4.	
Assumptions of land-use model	Lower crop yields	We proportionally decrease the expected yields of rice and maize. Timber and cattle yields remain unchanged.	Lowers NPV and food production and increases payback period of rice, maize and alley cropping. All other coefficients as per Table 4.	Simulates poorer site conditions, where lower yields from annual crops are expected. Sensitivity analysis in case yields in baseline scenario are too optimistic for the study area.
	Agroforestry subsidy	We proportionally decrease the investment costs of alley cropping and silvopasture.	Increases NPV and decreases payback period and investment costs of alley cropping and silvopasture. All other coefficients as per Table 4.	Simulates financial support from government programs to promote agroforestry establishment. For example, government agencies could provide free tree seedlings and/or fencing materials (for tree guards) to reduce the cost of establishing agroforestry.
	Higher timber prices	We proportionally increase the expected (baseline) timber price for teak and cedar.	Increases NPV and decreases payback period of alley cropping and teak plantation, increases NPV of silvopasture. All other coefficients as per Table 4.	Simulates favourable development of wood markets. Could also simulate tax exemptions on timber sales.

The scenarios can be divided into two groups: those that change the assumptions of the multi-criteria (optimisation) model, and those that change the assumptions of the land-use model

In the first set of scenarios, we retain the socio-economic coefficients from Table 4, and instead change the structure of the multi-criteria optimisation model. These scenarios therefore mimic different characteristics of the decision-maker. For instance, in the baseline scenario the five socio-economic indicators are weighted equally, but in the “Prioritising individual objectives” scenario we explore the impact of putting more weight on single indicators, to reflect the optimal portfolio for farmers with different priorities (Section 7.1 in Supplementary material details the weighting procedure). In the scenarios “Investment constraints and Labour constraints”, we impose fixed limits in the optimisation model to determine the optimal portfolio for farms with different labour or investment budgets. Moreover, we also tested these fixed limits when including farmers’ land-use preferences, as measured by Gosling et al. (2020a), as an additional indicator in the multi-criteria optimisation model (see Section 7.2 in the Supplementary material for details). These preferences may serve as a proxy for farmers’ cultural values (Knoke et al. 2014).

The second set of scenarios retain the baseline structure of the multi-criteria model (i.e., objectives weighted equally and no labour/investment constraints), and instead alter the assumptions and coefficients of the land-use model. These scenarios test environmental, market and political factors that are more external to the decision-maker. For example, in the “Lower crop yields” scenario we progressively decrease the expected yields of annual crops (rice and maize) within the monoculture and alley cropping systems, to simulate less productive soils and poorer growing conditions. In the scenario “Agroforestry subsidy”, we decrease the investment costs associated with silvopasture and alley cropping; here we simulate government subsidies or cost-sharing arrangements that reduce the tree establishment costs for farmers wishing to adopt agroforestry. Finally, in the “Higher timber prices” scenario we simulate favourable development of wood markets, progressively increasing the expected (baseline) price of teak and cedar.

For the second set of scenarios, all changes to the land-use model were made proportionally: we increased or decreased a variable by 0–100% in 10% steps. For each 10% change, we reran the Monte Carlo simulations to generate a new mean and standard deviation for the relevant land uses and indicators, and then reran the multi-criteria model with these new input data. We present the results for a high level of uncertainty (*m* = 3.0), based on the assumption that smallholder farmers are likely to be strongly risk-averse (Baker et al. 2017; Pannell et al. 2014), but the results for a lower level of risk aversion (*m* = 1.5) are also given in the Supplementary material (Fig. S6). The overall aim of the scenario analysis was to explore the conditions under which agroforestry becomes a more (or less) attractive land-use option for a risk-averse farmer.

## Results

### Baseline Scenario

**Fig. 2:**
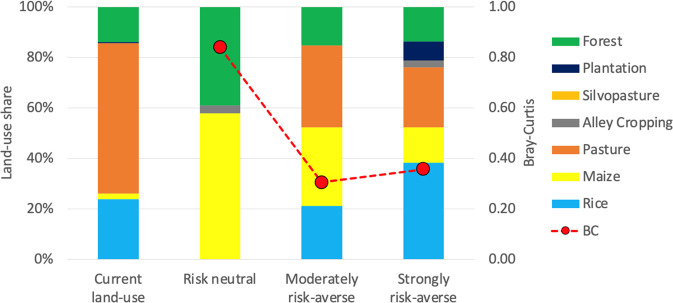
Composition of the optimised farm portfolio (share of land area allocated to each land use, left axis) for three levels of uncertainty: risk neutral (*m* = 0), moderately risk-averse (*m* = 1.5), and strongly risk-averse (*m* = 3.0) under the baseline scenario. The first column represents the current (aggregated) land use of the study area (data from Gosling et al. 2020a). Points represent the Bray–Curtis measure of dissimilarity (BC_*o,c*_, right axis) between the current and optimised land-use compositions: lower values indicate that a portfolio is more similar to the current land use

Figure 2 shows the optimal land-use composition for reducing trade-offs between the five socio-economic objectives under baseline conditions for the three levels of risk aversion. These optimal land-use compositions largely exclude agroforestry. Only alley cropping is selected in low (3%) shares: either to complement maize as a non-protective land-use when risk is disregarded, or as part of a diversification strategy at a high level of risk aversion.

According to the multi-criteria model, a risk neutral farmer (i.e., a farmer who disregards potential fluctuations in land-use performance) would allocate 58% of their land to maize, 3% to alley cropping and leave the rest as unmanaged forest (Fig. 2, second column from left). Maize dominates this farm portfolio because of its high predicted values for food production and NPV, while the large (39%) share of natural forest reduces the overall labour demand, investment costs and payback period of the portfolio. However, maize yields and prices vary quite strongly from year to year, making the maize monoculture a risky land use. Therefore at higher levels of risk aversion less maize is selected in the optimal portfolios, which become more diversified, also at the expense of protective land uses (natural forest). A moderately risk-averse farmer, for instance, would include a 33% share of pasture in their portfolio, reduce the maize share to 31% and supplement annual crop production with a 21% share of rice, leaving only 15% of the land as natural forest. A strongly risk-averse farmer would further diversify their land use with an 8% and 3% share of teak plantation and alley cropping, respectively. We therefore see that the optimal mix of land uses for achieving the five socio-economic objectives will depend on the decision-maker’s attitude toward risk. The two portfolios derived for a moderately and strongly risk-averse decision-maker are more similar to the current land-use allocation in the study area (leftmost column of Fig. 2) than the portfolio derived for a risk neutral farmer, as shown by the lower Bray–Curtis values.

### Accounting for Farmers’ Priorities, Preferences and Constraints

**Fig. 3:**
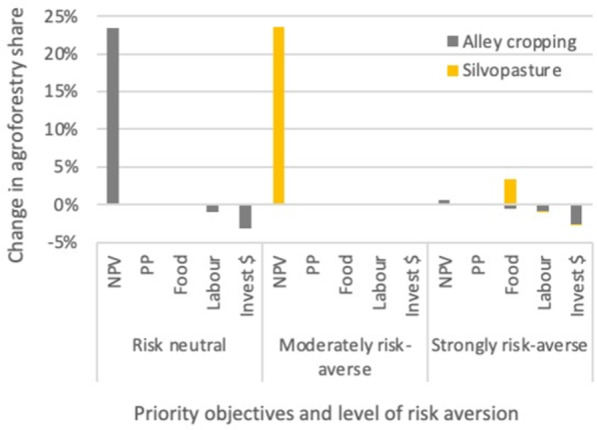
Relative change in the share of agroforestry selected in the optimal portfolio when prioritising one of the five indicators (net present value (NPV), payback periods (PP), Food production, Labour demand, Investment costs), for three levels of risk aversion. Prioritisation (weighting) method outlined in Table 5 and Section 7.1 of the Supplementary material

In the “Prioritising individual objectives” scenario, we found that giving higher weight to NPV strongly affects the type and share of agroforestry selected in the optimal portfolio. Weighting NPV as twice as important as the other indicators results in an optimal portfolio containing a substantial share of alley cropping (23%) for a risk neutral farmer (Fig. 3). A moderately risk-averse farmer would instead opt for 24% silvopasture. A very cautious decision-maker who prioritises NPV, however, would replace conventional pasture with annual crops in the optimal portfolio, with only a minimal increase in agroforestry. Prioritising the other indictors only had a minor impact on the share of agroforestry in the optimal portfolio.

An alternative method to account for farmers’ priorities would be to include their stated land-use preferences as an additional indicator in the multi-criteria model (see Section 7.2 of the Supplementary material). This approach favours the selection of agroforestry: the optimal portfolios that account for farmers’ stated land-use preferences contain a 11% and 21% share of silvopasture for a moderate and high level of risk aversion, respectively (Supplementary Fig. S3).

**Fig. 4:**
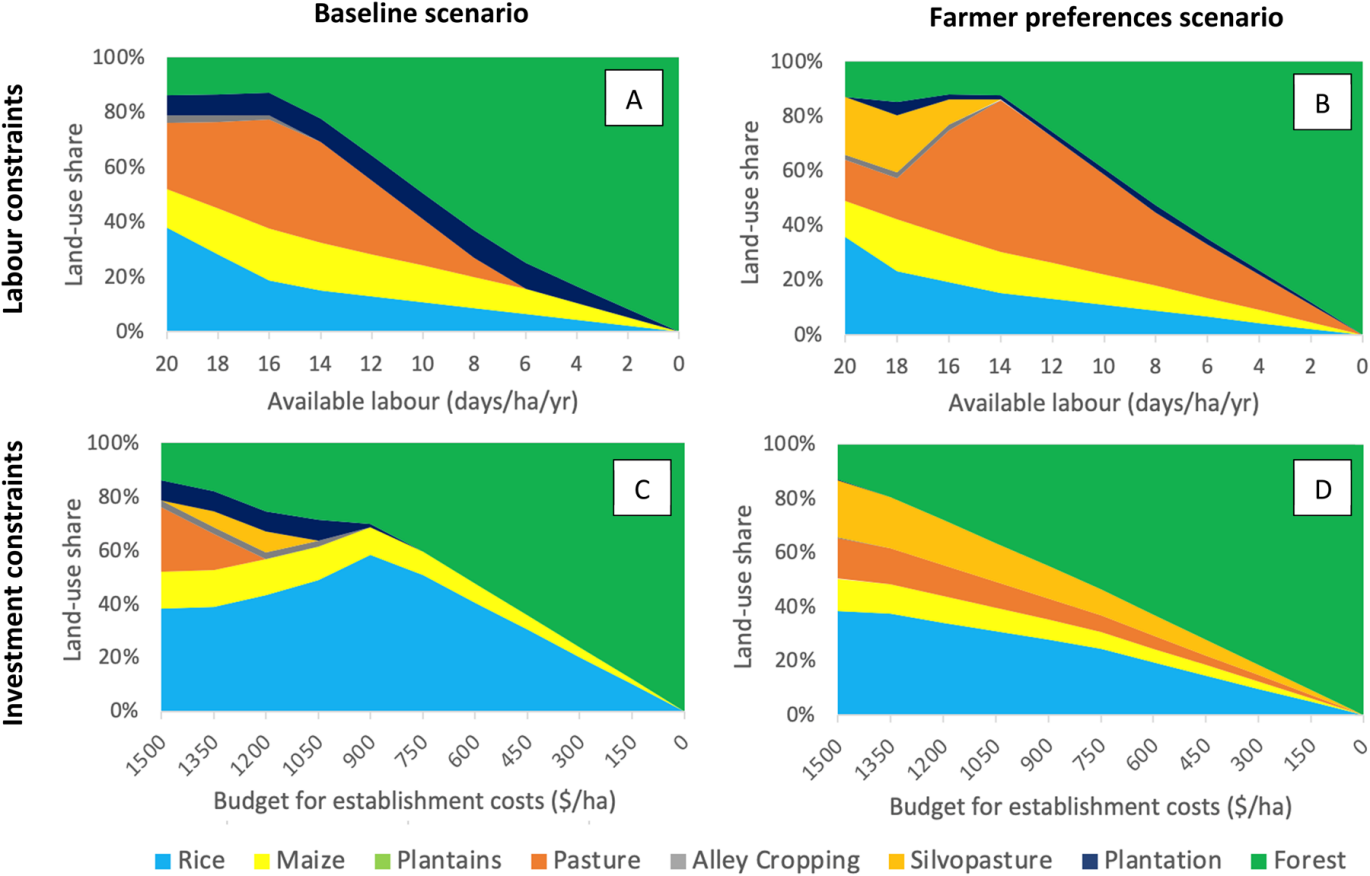
Composition of the ideal farm (share of land area allocated to each land-use option) for a strongly risk-averse farmer (*m* = 3.0), when imposing farm-level constraints in the “baseline” (plots A and C) and the “farmer preferences” scenarios (plots B and D), for which farmers’ general preferences are included as an additional indicator in the multi-criteria model (see Section 7.2 of the Supplementary material). In the plots A and B, the total amount of labour available to manage the land-use portfolio is progressively restricted. In plots C and D, the total investment budget for establishing the land-use portfolio is restricted

Taking the perspective now of a strongly risk-averse farmer, we see that the share of agroforestry in the optimal portfolio declined with increasing “Labour constraints” and “Investment constraints” (Fig. 4). However, we also see that agroforestry disappears more rapidly from the optimal portfolio under labour constraints than under investment constraints. This trend is especially clear when including farmers’ preferences as an additional indicator in the multi-criteria model, which increases the share of silvopasture in the constraint free portfolio.

For example, if labour is capped to less than 14 days per hectare per year, agroforestry could not compete with a mix of pasture, annual crops, teak plantation and forest (both under the baseline scenario and when considering farmer preferences: Fig. 4A, B). For a 50 ha farm, 2.3 workers would be needed to ensure 14 labour days are available per hectare per year^1^. As available labour continues to fall the share of productive land uses declines and forest cover increases (for both the baseline and farmer preference scenarios, Fig. 4A, B).

Decreasing the budget available for establishment costs initially leads to a small (6–8%) share of silvopasture in the optimal portfolio under the baseline scenario. But if a farmer cannot spend more than $1000 per hectare on land-use establishment, agroforestry is no longer included in the optimal portfolio (Fig. 4C). However, if farmers’ general preferences are also considered in the multi-criteria model (Fig. 4D), silvopasture is consistently included in the optimal portfolio even under severe budget constraints: in this scenario silvopasture always comprises around 21% of the non-protective land area (i.e., the land area not allocated to natural forest).

The fact that silvopasture persists in the optimal portfolio when restricting investment costs (Fig. 4D), but is quickly replaced with conventional pasture when imposing labour constraints (Fig. 4B), in part reflects the greater trade-off in labour demand compared to investment costs when switching from conventional pasture to silvopasture. For instance, conventional pasture already entails high investment costs ($1433 per hectare, 54% of which is used to purchase cattle), which in our land-use model are only 27% lower than those of silvopasture ($1970 per hectare, Table 4). In contrast, the difference in labour demand between the two cattle-based systems is more pronounced: conventional pasture saves 39% of the labour demand of silvopasture (pasture requires an average of 8 labour days per hectare per year compared to 14 labour days for silvopasture, Table 4). Therefore, as labour constraints increase, the model is more likely to select pasture over silvopasture (see, e.g., the increasing share of pasture in Fig. 4B).

### Simulating Changes in Environmental, Market and Political Conditions

**Fig. 5:**
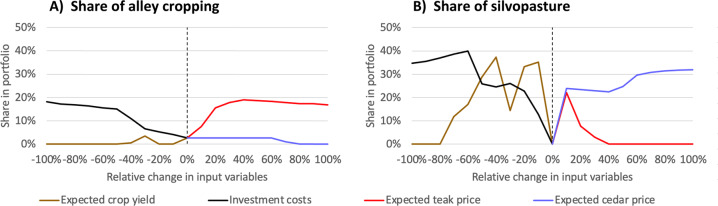
Share of **A** alley cropping and **B** silvopasture selected in the optimal land-use portfolio when changing the assumptions and coefficients of the land-use model. Input variables of the land-use model are progressively increased or decreased under three scenarios: changes to expected crop yields relate to the “lower crop yields” scenario, changes in investment costs to “agroforestry subsidy” and changes in teak and cedar price to “higher timber prices”. These scenarios are described in Table 5. Optimisation carried out from the perspective of a strongly risk-averse decision-maker (*m* = 3.0)

Figure 5 shows the relative change in the amount of agroforestry selected in the optimal portfolio when altering the assumptions and socio-economic coefficients of the land-use model. Across this group of scenarios, we see a stronger response of silvopasture than alley cropping; more silvopasture appears in the optimal portfolio. For example, silvopasture reached a maximum share of 40% when investment costs fell by 60% (black line, Fig. 5B). In contrast, the maximum share for alley cropping in the optimal portfolio was only 19%, achieved with a 40% increase in teak price (red line, Fig. 5A). We found a similar pattern of results for a moderately risk-averse farmer (Supplementary Fig. S6).

Simulating “Lower crop yields” (e.g., to find the optimal land allocation for a farm with less fertile soils) tends to favour the selection of silvopasture in the optimal portfolio. For example, silvopasture reached a share of 37% when expected crop yields declined by 40% (brown line in Fig. 5 B). Conversely, the share of alley cropping selected in the optimal portfolio fell to zero as expected crop yields declined (brown line in Fig. 5A).

Reducing investment costs under the “Agroforestry subsidy” scenario increased the share of both agroforestry systems in the optimal portfolio, but silvopasture to a greater extent. On average the alley cropping share increased by 1.6 percentage points per 10% drop in investment costs. In contrast, the share of silvopasture increased by two and a half times this rate (3.9 percentage points per 10% drop in investment costs). Providing farmers with tree seedlings and tree guards free of charge would reduce the total establishment costs of alley cropping and silvopasture by 20% and 13%, respectively. This would result in a 5% share of alley cropping and 20% share of silvopasture in the optimal portfolio (Supplementary Fig. S5).

Similarly, “Higher timber prices” promoted both agroforestry systems in the optimal portfolio, but silvopasture in particular. On average the share of alley cropping rose by 1.4 percentage points per 10% increase in teak price, whereas the silvopasture share rose by 3.5 percentage points per 10% increase in cedar price. Interestingly, the share of silvopasture initially increases with rising teak prices as silvopasture replaces pasture in the optimal portfolio (Supplementary Fig. S4d).

## Discussion

Agroforestry is not yet widespread in the study area, nor was it prominent in the optimised portfolio under baseline conditions. Here the similarity between the optimal portfolios for risk-averse farmers and the current land-use composition in Tortí (see Bray–Curtis values in Fig. 2) speaks for the plausibility of our model results. Given the Panamanian Government’s policy to increase agroforestry practices in rural areas (MiAmbiente 2019), it is vital to understand the factors that could help facilitate a transition from conventional to more tree-based farming systems among smallholders. Our modelling approach is well suited to this task, because it allows us to look beyond the current land-use composition to investigate theoretically optimal land allocations under different environmental or socio-economic conditions. This scenario analysis allows us to explore the factors that may promote or hinder the selection of agroforestry within a diversified land-use portfolio: an analysis that may prove extremely difficult when relying on empiric methods alone.

### Targeting Agroforestry: the Role of Farmer Priorities, Preferences and Attitudes toward Risk

Our model may help to understand the types of farmers for whom agroforestry may be most attractive, helping to target extension programs accordingly. For example, our “Prioritising individual objectives” scenario revealed large shares of agroforestry in the portfolios optimised for risk neutral and moderately risk-averse farmers who prioritise long-term income (quantified through NPV) over the other socio-economic objectives. This suggests that alley cropping and silvopasture may be attractive options for farmers who are more focused on longer-term profit but also more willing to accept risk. NPV could be an especially pertinent indicator for wealthy farmers, who may not depend as much on frequent and regular cash income from pastures or annual crops (Knoke et al. 2020b). The promotion of these agroforestry systems could therefore be targeted towards profit-oriented farmers managing larger farms, who have diversified income sources, including off-farm earnings, that help buffer financial risks (Bowman and Zilberman 2013).

Relying on NPV alone as a selling point for agroforestry, however, may limit the widespread adoption in regions where profit-oriented farmers are the exception rather than the rule. This may be the case in our study area. For instance, Gosling et al. (2020a) found that the shorter-term goals of maintaining liquidity and meeting subsistence needs (as opposed to long-term profit) could best explain farmers’ current land-use decisions in Tortí. Other studies in the tropics have also found that smallholder farmers tend to prioritise immediate needs related to cash flow and food security over long-term goals of profit maximisation (Affholder et al. 2010; Umar 2013). It is therefore vital to explore the conditions under which agroforestry can help achieve a broader set of farm-level goals.

It is promising that accounting for farmers’ stated land-use preferences as an additional indicator in the multi-criteria model (Fig. S3) enhanced the share of silvopasture in the optimised portfolio, because it suggests that this agroforestry system is compatible with farmers’ cultural values. In contrast, the lack of alley cropping in this portfolio implies that the silvoarable system may be less socially acceptable for farmers (despite being more profitable and less labour intensive than silvopasture). Cultural values can be important barriers or drivers of agroforestry adoption (Rahman et al. 2017; Tsonkova et al. 2014). Therefore, we would recommend developing and promoting silvopastoral (rather than silvoarable) systems in the study area, to better align with the cultural preferences of local farmers, recognising the importance of cattle for farmers’ livelihoods as a form of insurance and personal savings (Peterson St-Laurent et al. 2013). Nonetheless, demonstration farms that showcase alley cropping systems may help raise awareness and technical knowledge of this form of agroforestry among local farmers, which over time could foster greater acceptance of tree–crop systems within the farming community.

Farmers’ individual attitudes towards risk, however, will also influence the relative attractiveness of the two agroforestry options. In general, the highest shares of agroforestry occurred in portfolios optimised for a highly risk-averse farmer. This highlights the advantage of agroforestry as a diversification strategy to reduce risk (Baker et al. 2017; Lin 2011; Waldron et al. 2016). Across the different scenarios we found that land-use portfolios optimised for risk-averse farmers generally contained more silvopasture than alley cropping. This suggests that silvopasture may be the better option for avoiding underperformance of the socio-economic objectives under uncertainty, because it holds relatively low risks. Silvopasture offers the security of annual income from cattle sales, for which yields and prices are typically stable (Connelly and Shapiro 2006), with the bonus of additional income from cedar at the end of the rotation. In contrast, alley cropping cannot guarantee an annual income because shading restricts maize cultivation from year 3 onwards. Instead, the bulk of revenue flows rely on timber prices at three points of time (the two thinnings and final harvest), which makes it inherently risky. Paul et al. (2017) also report elevated risk levels for alley cropping compared to monoculture crops. Therefore, alley cropping may be less compatible with risk-averse decision-making.

### The Effect of Labour, Budget and Land Constraints

Despite farmers’ preference for silvopasture (Gosling et al. 2020a), this agroforestry system is not common practice in the study area. This may reveal a conflict between the land-use systems that farmers wish to have, and those that they are able to implement given their hard economic constraints (Gosling et al. 2020b; Tschakert et al. 2007). Expanding on previous studies (Gosling et al. 2020a, b), we explore the role of such farm-level restrictions on the optimal land-use composition by imposing fixed limits for labour demand and investment budgets in the optimisation model.

As expected, we found that “Labour and Investment constraints” reduce the share of agroforestry selected in the optimal portfolio. This aligns with other studies that found investment costs and labour demand to be barriers to agroforestry adoption in Latin America (e.g., Calle et al. 2009; Dagang and Nair 2003; Frey et al. 2012b). We found that silvopasture persists in the optimal portfolio when restricting investment costs, but is quickly replaced with conventional pasture when imposing labour constraints, suggesting that labour demand may pose the bigger barrier to silvopasture adoption.

In our model, the relative increase in labour demand when selecting silvopasture over conventional pasture is greater than the relative increase in investment costs, meaning the agroforestry system is hit harder by labour constraints. In practice, labour constraints may also be harder to overcome than capital constraints for farmers in the study area. It is common for farmers in Tortí to take out a loan to buy cattle when establishing conventional pasture systems (Peterson St-Laurent et al. 2013); the additional capital needed to establish trees for silvopastoral systems may be attainable through such loans, offering a means to overcome investment constraints. Meeting the additional labour requirement for silvopasture, however, may be more problematic, especially in tight labour markets (Baker et al. 2017; Pichón 1997). Labour shortages could be exacerbated by a hollowing of the forest frontier, which Sloan (2008) has already observed in eastern Panama: this is a phenomenon where the population density of a deforested landscape declines as extensive farming practices increase. Peterson St-Laurent et al. (2013) also report strong out-migration in eastern Panama as young people move to cities. In the face of tight labour markets it may therefore be necessary to adapt silvopastoral systems to better meet the needs of farmers constrained by labour shortages. This could be done by improving economies of scope, for example, through the use of multi-purpose trees where pruning could be combined with fodder production (Reyes Cáceres 2018). Such economies of scope are already a key advantage of the alley cropping system, in which trees and crops are weeded simultaneously (Paul et al. 2017).

Farmers’ land-use decisions will also be constrained by site conditions, which will influence the relative attractiveness of agroforestry. For example, simulating “Lower crop yields” increased the share of silvopasture selected in the optimal portfolio of a risk-averse decision-maker. This suggests that silvopasture may be a more attractive land-use option for farmers with less productive land (on which it is not possible to cultivate high yielding crops). These findings align with bio-economic studies that suggest agroforestry may be more advantageous on poorer growing sites (Crestani et al. 2017; Tsonkova et al. 2014). Moreover, the results underline the general importance of land condition (i.e., soil type and quality) for influencing the uptake of agroforestry and agricultural innovations (Pannell et al. 2014; Pattanayak et al. 2003).

### Subsidies and Timber Prices to Promote Agroforestry Adoption

We found that the selection of agroforestry in the optimal portfolio was most responsive to a potential “Agroforestry subsidy” (lowering investment costs) and “Higher timber prices”. This suggests that cost-sharing arrangements could be an effective strategy to boost agroforestry adoption in the study area. For example, providing farmers with free tree seedlings and tree guards resulted in a 5 and 20% share of alley cropping and silvopasture in the optimal portfolio. Given its higher labour demand compared to conventional pasture, greater adoption of silvopasture could generate employment opportunities in the region if farmers hire day workers to assist with tree planting and pruning (Frey et al. 2012a). Establishment grants for silvopasture could help farmers finance this additional labour. While the legal framework for such incentives exists, they are yet to be consistently implemented in the study area.

In our scenario testing, we found that moderate increases in timber prices could lead to substantial shares of agroforestry being selected in a land-use portfolio that balances trade-offs between the five socio-economic objectives. For example, a 30% increase in teak price would result in a 18% share of alley cropping in the optimal portfolio, while a 30% increase in the cedar price would lead to a 33% silvopasture share. We also found that a small (10%) increase in the teak price could favour the selection of silvopasture in the portfolio. As the rising teak price makes alley cropping and plantation more profitable, the underperformance of pasture in terms of NPV becomes too great and it is first replaced with silvopasture and then by alley cropping and teak plantation in the optimal portfolio as the teak price continues to increase (Supplementary Fig. S4d).

Timber prices strongly depend on market factors, and are thereby harder to engineer through government programs. However, the Panamanian Government’s recently legislated tax exemptions for timber grown in agroforestry systems (Law 69, 2017) could increase revenues from timber sales. Such tax incentives could particularly benefit the selection of alley cropping, which would become more competitive against pure teak plantation. This assumes, however, that farmers are earning enough to pay income tax, which may not be the case for many farm households (Díaz et al. 2012). Alternatively, farmer training programs on tree management (e.g., pruning and pest control techniques) could improve silvicultural practices, helping farmers to produce higher quality timber and hence obtain higher prices. Training programs and certification schemes could also help farmers build their capacity to access markets and obtain price premiums (Holmes et al. 2017; Somarriba et al. 2012). Nonetheless, when considering current timber prices (baseline scenario), only very small shares of agroforestry were included in the optimal portfolio. This could signal that further development of timber markets is a prerequisite for widespread adoption of timber-based land-use systems among smallholder farmers in the study area.

### Limitations of Modelling Approach and Research Outlook

Our study is a rare example of a multi-criteria evaluation of agroforestry that takes a portfolio approach to account for the effects of land-use diversification and uncertainty on farmers’ land-use decisions. However, we acknowledge limitations of our study, which could be addressed in future research.

First, we rely on static modelling approaches in both the land-use and multi-criteria models. For instance, the land-use model ignores adverse environmental effects such as soil depletion over time (Janssen and van Ittersum 2007). This may overestimate the productivity of conventional land uses, and hence downplay drivers of agroforestry adoption. Future studies could therefore integrate production decay functions (e.g., following Sanchez 1976) to better account for the effect of nutrient depletion and soil structural changes on crop yields. Similarly, the multi-criteria model identifies theoretically optimal land allocations, but not how these could be achieved over time. Using a more dynamic optimisation approach, such as the one Knoke et al. (2020a) recently developed to investigate smallholders’ deforestation decisions in Ecuador, would allow us to simulate farmers’ land-use decisions in smaller time steps. This would allow for staggered planting of trees, which might be a more feasible path for smallholders to adopt agroforestry (Bertomeu and Giménez 2006). A dynamic approach may also help to account for the option value of agroforestry systems and their conventional counterparts, an aspect which is overlooked in this study. In our land-use model, the timing of timber harvesting is fixed: this fails to capture the flexibility that a farmer has to postpone harvest if timber prices are unfavourable (Frey et al. 2013).

Second, our robust optimisation model is not spatially explicit. The model identifies what portions of a hypothetical farm could be allocated to each land-use option, but does not specify the exact location or arrangement of these land-use options (Bertomeu and Giménez 2006). This approach implicitly assumes homogeneous site conditions. Therefore, our multi-criteria model ignores the potential influence that farmers’ existing land use as well as variation in soil quality, slope and distance from the farm homestead may have on their land-use decisions, including their adoption of agroforestry (Bannister and Nair 2003; Pannell et al. 2014; Pattanayak et al. 2003). Thus, caution is needed when generalising the model results to farms with highly heterogeneous soils and/or contrasting topography, both within and outside of the study area.

Third, we integrated tree–crop and tree–pasture interactions in our land-use model through plausible assumptions (Paul et al. 2015), rather than detailed biophysical modelling. Our projected tree growth and crop yields were comparable to those simulated for the study area using the tree–crop model WaNuLCAS (Paul et al. 2017), while the economic coefficients for pasture-based systems reflect the lower, but very stable economic returns of cattle grazing in Panama (Connelly and Shapiro 2006). Nevertheless, the modelling approach could be enhanced by integrating biophysical modelling to simulate tree, crop and pasture growth in monoculture and agroforestry systems (e.g., using WaNuLCAS, Santos Martin and van Noordwijk 2011). Such modelling could be particularly useful for evaluating different layouts of agroforestry systems, for example, to identify the most promising systems for field trials. Ultimately, such local field experiments are essential to obtain empiric data, which remains the best foundation for land-use planning (Reith et al. 2020).

In presenting our study, we recognise the usefulness, but also limits, of models as decision support tools. Our modelling approach explores theoretically optimal land allocations for achieving a particular outcome under a certain set of assumptions. We do not intend to prescribe exact farm compositions that farmers in the study area should adhere to. Instead, we seek to explore the conditions under which agroforestry might be a desirable complement to help farmers reduce trade-offs between socio-economic objectives. The decision of whether or not to adopt a given land-use system rests with the farmer, and will depend on his or her objectives and constraints (Janssen and van Ittersum 2007; Pannell et al. 2006). Our study therefore does not seek to develop a decision support tool for farmers, but is rather targeted at researchers and political decision-makers. For researchers our modelling approach may help to identify the agroforestry systems and conditions under which more detailed field trials are most warranted, because the systems show a high probability of being of interest to farmers. For policy-makers, such approaches can help to identify the circumstances under which promoting agroforestry appears to be promising without generating conflicts with farmers’ goals.

However, as with any decision support tool, we acknowledge a potential gap between the results of our theoretical model and the reality of farmers’ decision-making (McCown 2001). Such gaps between theory and practice may stem from potential biases and uncertainties in model input data. Although we actively account for such uncertainty by implementing a form of robust optimisation (Doole 2012; Knoke et al. 2015), field experiments remain crucial to deliver reliable empiric data. The gap between theory and practice may also stem from the assumptions and limitations of the multi-criteria model, which cannot capture all aspects influencing farmers’ decisions. For example, in the scenario analysis we alter one aspect at a time to understand how this affects the share of agroforestry selected in the optimal portfolio. In reality, however, such aspects will be changing simultaneously, potentially leading to complex interactions that we do not account for. With these limitations in mind, care is needed when generalising our results to other areas: the more the region differs to the biophysical and socio-economic conditions of Tortí, the greater the gap is likely to be between our theoretical and the actually optimal land allocations. However, we again emphasise that we do not seek to give exact land-use recommendations for this study site, but rather demonstrate how such an approach may inform future research and policy design.

Finally, we see potential to further develop our approach through participatory and collaborative modelling. Indeed, greater farmer interaction is likely to help narrow the gap between scientific theory and real-world practice (Janssen and van Ittersum 2007; McCown 2001). For example, farmers could help to validate input data, based on their local knowledge and experience. Moreover, as simple, stylised land-use portfolios, we believe the output of the multi-criteria model could be readily interpreted and evaluated by smallholder farmers. Discussing model results with farmers in the study area could help to validate and improve the model, for example, by changing objectives or adding additional constraints to better match the local situation (Groot et al. 2012). Optimised portfolios might also provide a good starting point for stakeholder discussions as part of participatory land-use planning (Le Gal et al. 2013). For this type of landscape-scale planning the multi-criteria model could easily integrate ecological indicators (either based on expert opinion, e.g. Reith et al. 2020, or modelled and measured data, e.g. Knoke et al. 2020a), to derive the optimal land-use compositions for achieving a wider range of ecosystem services.

## Conclusion

Insights gained through our modelling approach can help to identify socially acceptable agroforestry systems for on-farm trials, and to design effective and efficient incentive and extension programs. For our case study in eastern Panama, we found that silvopasture may be most suited for meeting the needs of a risk-averse farmer, given the frequent and stable returns from cattle and the compatibility of this system with local farmers’ cultural values. Poorer growing conditions for annual crop are likely to enhance the attractiveness of silvopasture as a land-use option, as would government support to subsidise tree-planting costs. However, the uptake of silvopasture may be limited on farms where less labour is available. Despite being the more profitable agroforestry system, we found that alley cropping was less compatible with farmers’ cultural values and risk aversion. This system may nonetheless be a suitable complement to a diversified farm portfolio for more risk-tolerant, profit-oriented farmers. While we present an example from a tropical forest frontier region, the multi-criteria optimisation method is transferable to investigate sustainable land-use systems in other agricultural or forested landscapes.

## Supplementary Information

Supplementary Material

## Data Availability

All data sources used are appropriately cited. Original model files are available from the authors upon request.

## Footnotes

1 This equates to 0.05 workers per hectare. By comparison, the average labour availability of farms interviewed by Gosling et al. (2020a) was 0.08 workers per hectare.

## References

[CR1] Affholder F, Jourdain D, Quang DD, Tuong TP, Morize M, Ricome A (2010). Constraints to farmers’ adoption of direct-seeding mulch-based cropping systems: a farm scale modeling approach applied to the mountainous slopes of Vietnam. Agric Syst.

[CR2] Aldea J, Martínez-Peña F, Romero C, Diaz-Balteiro L (2014). Participatory goal programming in forest management: an application integrating several ecosystem services. Forests.

[CR3] ANAM (2011) Atlas Ambiental de la República de Panamá, Primera Versión. Autoridad Nacional del Ambiente (ANAM). Gobierno Nacional República de Panamá, Ciudad de Panamá

[CR4] Andrade HJ, Brook R, Ibrahim M (2008). Growth, production and carbon sequestration of silvopastoral systems with native timber species in the dry lowlands of Costa Rica. Plant Soil.

[CR5] Baker K, Bull G, Baylis K, Barichello R (2017). Towards a theoretical construct for modelling smallholders’ forestland-use decisions: what can we learn from agriculture and forest economics?. Forests.

[CR6] Ballarin A, Vecchiato D, Tempesta T, Marangon F, Troiano S (2011). Biomass energy production in agriculture: a weighted goal programming analysis. Energy Policy.

[CR7] Bannister ME, Nair PKR (2003). Agroforestry adoption in Haiti: the importance of household and farm characteristics. Agrofor Syst.

[CR8] Baumgärtner S, Quaas MF (2010). Managing increasing environmental risks through agrobiodiversity and agrienvironmental policies. Agric Econ.

[CR9] Bertomeu M, Giménez JC (2006). Improving adoptability of farm forestry in the Philippine uplands: a linear programming model. Agrofor Syst.

[CR10] Binh DKNT, Phuong LTV, Douglas I, van De N, McMorrow J, Lindley S, Van TT, Thanh LH, Tho N (2008). Local knowledge and economic realities affecting soil erosion in the Rach Rat Catchment, Vietnam. Geogr Res.

[CR11] Biswas A, Pal BB (2005). Application of fuzzy goal programming technique to land use planning in agricultural system. Omega.

[CR12] Blandon P (2005) Analyzing risk in agroforestry systems using a portfolio approach. In: Alavalapati JRR, Mercer DE (eds) Valuing agroforestry systems: methods and applications, vol 2. Springer + Business Media Inc., Dordrecht, p 95–122

[CR13] Bowman MS, Zilberman D (2013) Economic factors affecting diversified farming systems. Eol Soc 18. 10.5751/ES-05574-180133

[CR14] Calle A, Montagnini F, Zuluaga AF (2009). Farmer’s perceptions of silvopastoral system promotion in Quindío, Colombia. Bois For Trop.

[CR15] Connelly A, Shapiro EN (2006). Smallholder agricultural expansion in La Amistad Biosphere Reserve. J Sustain For.

[CR16] Coomes OT, Grimard F, Potvin C, Sima P (2008). The fate of the tropical forest: carbon or cattle?. Ecol Econ.

[CR17] Cordero J, Boshier DH (eds) (2003) Árboles de Centroamérica: Un manual para extensionistas (Trees of Central America: a manual for extentionsts). CATIE, Turrialba, Costa Rica

[CR18] Corman JR, Collins SL, Cook EM, Dong X, Gherardi LA, Grimm NB, Hale RL, Lin T, Ramos J, Reichmann LG, Sala OE (2019). Foundations and frontiers of ecosystem science: legacy of a classic paper (Odum 1969). Ecosystems.

[CR19] Crestani S, Mascheroni JDC, Vera Geremia E, Carnevalli RA, Mourão GB, Da Silva SC (2017). Sward structural characteristics and herbage accumulation of Piatã palisade grass (Brachiaria brizantha) in a crop–livestock–forest integration area. Crop Pasture Sci.

[CR20] Dagang ABK, Nair PKR (2003). Silvopastoral research and adoption in Central America: recent findings and recommendations for future directions. Agrofor Syst.

[CR22] Diaz-Balteiro L, Romero C (2008). Making forestry decisions with multiple criteria: a review and an assessment. For Ecol Manag.

[CR99] Díaz I, Pineda, E, Arcia DI (2012) Incentivos y desincentivos a la actividad forestal en Panamá, Centro Agronómico Tropical de Investigación y Enseñanza (CATIE) http://repositorio.bibliotecaorton.catie.ac.cr/handle/11554/8738 Accessed 17 Nov 2020

[CR21] Di Falco S, Perrings C (2005). Crop biodiversity, risk management and the implications of agricultural assistance. Ecol Econ.

[CR23] Do H, Luedeling E, Whitney C (2020). Decision analysis of agroforestry options reveals adoption risks for resource-poor farmers. Agron Sustain Dev.

[CR24] Doole GJ (2012). Evaluation of an agricultural innovation in the presence of severe parametric uncertainty: an application of robust counterpart optimisation. Comput Electron Agric.

[CR25] Duarte R (2018) Relating land-use diversification to household (socio-economic) characteristics in Eastern Panama. Bachelor thesis, Technical University of Munich, Freising, Germany

[CR26] ETESA (2018) Historical data on mean annual rainfall in Tortí (1977-2018). www.hidromet.com.pa/clima_historicos.php. Accessed 7 Jan 2019

[CR27] FAO (2019) Technical conversion factors for agricultural commodities. www.fao.org/economic/the-statistics-division-ess/methodology/methodology-systems/technical-conversion-factors-for-agricultural-commodities/en. Accessed 14 Aug 2019

[CR28] Fassola HE, Lacorte SM, Pachas N, Pezzutti R (2006). Efecto de distintos niveles de sombra del dosel de *Pinus taeda L*. sobre la acumulación de biomasa forrajera de Axonopus compressus (Swartz) Beauv. Rev Argent Prod Anim.

[CR29] Fischer A, Vasseur L (2000). The crisis in shifting cultivation practices and the promise of agroforestry: a review of the Panamanian experience. Biodivers Conserv.

[CR30] Fischer A, Vasseur L (2002). Smallholder perceptions of agroforestry projects in Panama. Agrofor Syst.

[CR32] Frey GE, Fassola HE, Pachas AN, Colcombet L, Lacorte SM, Pérez O, Renkow M, Warren ST, Cubbage FW (2012). Perceptions of silvopasture systems among adopters in northeast Argentina. Agric Syst.

[CR31] Frey GE, Fassola HE, Pachas AN, Colcombet L, Lacorte SM, Renkow M, Perez O, Cubbage FW (2012). A within-farm efficiency comparison of silvopasture systems with conventional pasture and forestry in northeast Argentina. Land Econ.

[CR33] Frey GE, Mercer DE, Cubbage FW, Abt RC (2013). A real options model to assess the role of flexibility in forestry and agroforestry adoption and disadoption in the Lower Mississippi Alluvial Valley. Agric Econ.

[CR34] García-de Ceca J, Gebremedhin KG (1991). A decision support system for planning agroforestry systems. For Ecol Manag.

[CR98] García M, Vides C, Aguilar A, Vivar P (2016) Bonn Challenge Latin America, 2016 Report. Deutsche Gesellschaft für Internationale Zusammenarbeit (GIZ) https://www.iucn.org/news/forests/201612/outcomes-2016-latin-america-meeting-bonn-challenge. Accessed 17 Nov 2020

[CR35] Gardi C, Angelini M, Barceló S, Comerma J, Cruz Gaistardo C, Encina Rojas A, Jones A, Krasilnikov P, Mendonça Santos Brefin ML, Montanarella L, Muñiz Ugarte O, Schad P, Vara Rodríguez MI, Vargas R, Ravina da Silva M (eds) (2015) Soil atlas of Latin America and the Caribbean. EUR, 25402 EN. European Commission—Publications Office of the European Union, L-2995 Luxembourg

[CR37] Gosling E, Reith E, Knoke T, Gerique A, Paul C (2020b) Exploring farmer perceptions of agroforestry via multi-objective optimisation: a test application in Eastern Panama. Agrofor Syst 94:2003–2020. 10.1007/s10457-020-00519-0

[CR36] Gosling E, Reith E, Knoke T, Paul C (2020). A goal programming approach to evaluate agroforestry systems in Eastern Panama. J Environ Manage.

[CR38] Griess VC, Knoke T (2011). Can native tree species plantations in Panama compete with teak plantations?: an economic estimation. New For.

[CR39] Groot JCJ, Oomen GJM, Rossing WAH (2012). Multi-objective optimization and design of farming systems. Agric Syst.

[CR40] Holmes I, Kirby KR, Potvin C (2017). Agroforestry within REDD+: experiences of an indigenous Emberá community in Panama. Agrofor Syst.

[CR41] INEC (2011) Censo Nacional Agropecuario 2010, VIII Tenencia y Aprovechamiento de la Tierra. Explotaciones. Instituto Nacional de Estadística y Censo (INEC). https://www.contraloria.gob.pa/inec/Publicaciones/subcategoria.aspx?ID_CATEGORIA=15&ID_SUBCATEGORIA=60&ID_IDIOMA=1ID_SUBCATEGORIA=60&ID_PUBLICACION=470&ID_IDIOMA=1&ID_CATEGORIA=15. Accessed 14 Aug 2019

[CR42] Janssen S, van Ittersum MK (2007). Assessing farm innovations and responses to policies: a review of bio-economic farm models. Agric Syst.

[CR43] Jose S (2009). Agroforestry for ecosystem services and environmental benefits: an overview. Agrofor Syst.

[CR44] Jose S, Walter D, Mohan Kumar B (2017). Ecological considerations in sustainable silvopasture design and management. Agrofor Syst.

[CR45] Kaim A, Cord AF, Volk M (2018). A review of multi-criteria optimization techniques for agricultural land use allocation. Environ Model Softw.

[CR46] Knoke T, Bendix J, Pohle P, Hamer U, Hildebrandt P, Roos K, Gerique A, Sandoval ML, Breuer L, Tischer A, Silva B, Calvas B, Aguirre N, Castro LM, Windhorst D, Weber M, Stimm B, Günter S, Palomeque X, Mora J, Mosandl R, Beck E (2014). Afforestation or intense pasturing improve the ecological and economic value of abandoned tropical farmlands. Nat Commun.

[CR51] Knoke T, Gosling E, Paul C (2020b) Use and misuse of the net present value in environmental studies. Ecol Econ 174:106664. 10.1016/j.ecolecon.2020.106664

[CR47] Knoke T, Paul C, Härtl F, Castro LM, Calvas B, Hildebrandt P (2015). Optimizing agricultural land-use portfolios with scarce data—a non-stochastic model. Ecol Econ.

[CR48] Knoke T, Paul C, Hildebrandt P, Calvas B, Castro LM, Härtl F, Döllerer M, Hamer U, Windhorst D, Wiersma YF, Curatola Fernández GF, Obermeier WA, Adams J, Breuer L, Mosandl R, Beck E, Weber M, Stimm B, Haber W, Fürst C, Bendix J (2016). Compositional diversity of rehabilitated tropical lands supports multiple ecosystem services and buffers uncertainties. Nat Commun.

[CR50] Knoke T, Paul C, Rammig A, Gosling E, Hildebrandt P, Härtl F, Peters T, Richter M, Diertl K-H, Castro LM, Calvas B, Ochoa S, Valle-Carrión LA, Hamer U, Tischer A, Potthast K, Windhorst D, Homeier J, Wilcke W, Velescu A, Gerique A, Pohle P, Adams J, Breuer L, Mosandl R, Beck E, Weber M, Stimm B, Silva B, Verburg PH, Bendix J (2020a) Accounting for multiple ecosystem services in a simulation of land-use decisions: does it reduce tropical deforestation? Global Change Biology. 10.1111/gcb.15003 10.1111/gcb.15003 31957121

[CR49] Knoke T, Messerer K, Paul C (2017). The role of economic diversification in forest ecosystem management. Curr For Rep.

[CR52] Laroche G, Domon G, Gélinas N, Doyon M, Olivier A (2018). Integrating agroforestry intercropping systems in contrasted agricultural landscapes: a SWOT-AHP analysis of stakeholders’ perceptions. Agrofor Syst.

[CR55] Leakey RRB (2020). A re-boot of tropical agriculture benefits food production, rural economies, health, social justice and the environment. Nat Food.

[CR54] Le Gal P-Y, Bernard J, Moulin C-H (2013). Supporting strategic thinking of smallholder dairy farmers using a whole farm simulation tool. Trop Anim Health Prod.

[CR53] Le Gal P-Y, Dugué P, Faure G, Novak S (2011). How does research address the design of innovative agricultural production systems at the farm level?: a review. Agric Syst.

[CR56] Lin BB (2011). Resilience in agriculture through crop diversification: adaptive management for environmental change. BioScience.

[CR57] Liu W, Yao S, Wang J, Liu M (2019). Trends and features of agroforestry research based on bibliometric analysis. Sustainability.

[CR58] Markowitz H (1952). Portfolio selection. J Finance.

[CR59] McCown RL (2001). Learning to bridge the gap between science-based decision support and the practice of farming: evolution in paradigms of model-based research and intervention from design to dialogue. Aust J Agric Res.

[CR60] Mendoza GA, Campbell GE, Rolfe GL (1987). Multiple objective programming: an approach to planning and evaluation of agroforestry systems: part 2—an illustrative example and analysis. Agric Syst.

[CR61] Messerer K, Pretzsch H, Knoke T (2017). A non-stochastic portfolio model for optimizing the transformation of an even-aged forest stand to continuous cover forestry when information about return fluctuation is incomplete. Ann For Sci.

[CR62] MiAmbiente (2019) Alianza por el Millón REDD+ de Panamá. Ministerio de Ambiente de Panamá (MiAmbiente) https://www.pa.undp.org/content/panama/es/home/library/environment_energy/alianza-por-el-millon-redd-.html. Accessed 17 Nov 2020

[CR63] MIDA (2016). Direccion de ganaderia: Costo de 1 kilometro de cerca perimetral. November 2016.

[CR64] MIDA (2019a) Direccion de agricultura: Costo de produccion normativo de una hectarea de maiz. Ministerio de Desarrollo Agropecuario (MIDA). https://www.mida.gob.pa/direcciones/direcciones_nacionales/direcci-n-de-agricultura/costos-de-producci-n-2019.html. Accessed 12 Aug 2019

[CR65] MIDA (2019b) Direccion de agricultura: Costo de produccion normativo de una hectarea de arroz. Ministerio de Desarrollo Agropecuario (MIDA). https://www.mida.gob.pa/direcciones/direcciones_nacionales/direcci-n-de-agricultura/costos-de-producci-n-2019.html. Accessed 12 Aug 2019

[CR66] Montagnini F, Metzel R (2018) The contribution of agroforestry to Sustainable Development Goal 2: end hunger, achieve food security and improved nutrition, and promote sustainable agriculture. In: Montagnini F (ed) Integrating Landscapes. Springer, Cham, p 11–45

[CR67] Montambault JR, Alavalapati JRR (2005). Socioeconomic research in agroforestry: a decade in review. Agrofor Syst.

[CR68] Ochoa MWS, Paul C, Castro LM, Valle L, Knoke T (2016). Banning goats could exacerbate deforestation of the Ecuadorian dry forest—how the effectiveness of conservation payments is influenced by productive use options. Erdkunde.

[CR69] Odum EP (1969). The strategy of ecosystem development. Science.

[CR70] ONF (2019) Precios de la madera: Para las especies mas comercializadas. Primer semestre del 2019. Oficina Nacional Forestal de Costa Rica (ONF) https://onfcr.org/informe-de-precios-de-madera/. Accessed 14 Aug 2019

[CR71] Palma J, Graves AR, Burgess PJ, van der Werf W, Herzog F (2007). Integrating environmental and economic performance to assess modern silvoarable agroforestry in Europe. Ecol Econ.

[CR73] Pannell DJ, Llewellyn RS, Corbeels M (2014). The farm-level economics of conservation agriculture for resource-poor farmers. Agric Ecosyst Environ.

[CR72] Pannell DJ, Marshall GR, Barr N, Curtis A, Vanclay F, Wilkinson R (2006). Understanding and promoting adoption of conservation practices by rural landholders. Aust J Exp Agric.

[CR74] Pattanayak SK, Evan Mercer D, Sills E, Yang J-C (2003). Taking stock of agroforestry adoption studies. Agrofor Syst.

[CR75] Paul C (2014) Timber-based agrisilvicultural systems to facilitate reforestation in Panama: a silvicultural and economic evaluation. Dissertation, Technical University of Munich, Freising, Germany

[CR76] Paul C, Griess VC, Havardi-Burger N, Weber M (2015). Timber-based agrisilviculture improves financial viability of hardwood plantations: a case study from Panama. Agrofor Syst.

[CR77] Paul C, Weber M, Knoke T (2017). Agroforestry versus farm mosaic systems—comparing land-use efficiency, economic returns and risks under climate change effects. Sci Total Environ.

[CR78] Pearce D, Putz FE, Vanclay JK (2003). Sustainable forestry in the tropics: Panacea or folly?. For Ecol Manag.

[CR79] Peterson St-Laurent G, Gélinas N, Potvin C (2013). REDD+ and the agriculture frontier: understanding colonists’ utilization of the land. Land Use Policy.

[CR80] Pichón FJ (1997). Colonist land‐allocation decisions, land use, and deforestation in the Ecuadorian Amazon frontier. Econ Dev Cult Change.

[CR81] Rahman SA, Jacobsen JB, Healey JR, Roshetko JM, Sunderland T (2017). Finding alternatives to swidden agriculture: does agroforestry improve livelihood options and reduce pressure on existing forest?. Agrofor Syst.

[CR82] Reith E, Gosling E, Knoke T, Paul C (2020). How much agroforestry is needed to achieve multifunctional landscapes at the forest frontier?: coupling expert opinion with robust goal programming. Sustainability.

[CR83] Reyes Cáceres A (2018) Assessing the economic potential of agroforestry systems in Tortí, Panama. Master’s thesis, Technical University of Munich, Freising, Germany

[CR84] Romero C (2001). Extended lexicographic goal programming: a unifying approach. Omega.

[CR85] Sanchez PA (1976). Properties and management of soils in the tropics.

[CR86] Santos Martin F, van Noordwijk M (2011). Is native timber tree intercropping an economically feasible alternative for smallholder farmers in the Philippines?. Aust J Agric Resour Econ.

[CR87] Schuchmann J (2011) A participatory survey on current integration of trees on farms and pastures within land use systems in the township of Tortí in Panama. Bachelor thesis, Technical University of Munich, Freising, Germany

[CR88] Sloan S (2008). Reforestation amidst deforestation: simultaneity and succession. Glob Environ Change.

[CR89] Somarriba E, Beer J, Alegre-Orihuela J, Andrade HJ, Cerda R, DeClerck F, Detlefsen G, Escalante M, Giraldo LA, Ibrahim M, Krishnamurthy L, Mena-Mosquera VE, Mora-Degado JR, Orozco L, Scheelje M, Campos JJ (2012) Mainstreaming agroforestry in Latin America. In: Nair PR, Garrity D (eds) Agroforestry—the future of global land use. Springer Netherlands, Dordrecht, p 429–473

[CR90] Tschakert P, Coomes OT, Potvin C (2007). Indigenous livelihoods, slash-and-burn agriculture, and carbon stocks in Eastern Panama. Ecol Econ.

[CR91] Tsonkova P, Quinkenstein A, Böhm C, Freese D, Schaller E (2014). Ecosystem services assessment tool for agroforestry (ESAT-A): an approach to assess selected ecosystem services provided by alley cropping systems. Ecol Indic.

[CR92] Uhde B, Hahn WA, Griess VC, Knoke T (2015). Hybrid MCDA methods to integrate multiple ecosystem services in forest management planning: a critical review. Environ Manag.

[CR93] Umar BB (2013). A critical review and re-assessment of theories of smallholder decision-making: a case of conservation agriculture households, Zambia. Renew Agric Food Syst.

[CR94] USDA (2019) Food Composition Databases of the United States Department of Agriculture Agricultural Research Service. https://ndb.nal.usda.gov/ndb. Accessed 14 Aug 2019

[CR95] van Zonneveld M, Turmel M-S, Hellin J (2020). Decision-making to diversify farm systems for climate change adaptation. Front Sustain Food Syst.

[CR96] Waldron A, Garrity D, Malhi Y, Girardin C, Miller DC, Seddon N (2016). Agroforestry can enhance food security while meeting other sustainable development goals. Trop Conserv Sci.

[CR97] Walker WE, Lempert RJ, Kwakkel JH (2013) Deep Uncertainty. In: Gass SI, Fu M (eds) Encyclopedia of operations research and management science. Springer US, Boston, MA, p 395–402

