# Supplementary material for: Which Socio-economic Conditions Drive the Selection of Agroforestry at the Forest Frontier?
Source: Environ Manage. 2021 Feb 12;67(6):1119–36. doi: 10.1007/s00267-021-01439-0 (PMC8106585; doi:10.1007/s00267-021-01439-0)
Supplement: Supplementary file 1 — Supplementary Material [file 267_2021_1439_MOESM1_ESM.pdf]

**Supplementary Material**

## 1. Production costs

### *1.1. Cultivation of agricultural crops*

The production costs of rice and maize are based on information from the Ministry of Agricultural Development of Panama *Ministerio de Desarrollo Agropecuario de Panamá, MIDA:* (MIDA 2019a, 2019b). The annual cultivation costs remain constant over the 20-year period, except more labour is needed in the first year of cultivation to clear the site of secondary vegetation (Table S1).

**Table S1** Production costs for rice and maize monocultures, based on information for traditional (non-mechanised) systems with use of pesticides and fertilisers (MIDA 2019a, 2019b).

| | **Rice cultivation** | | **Maize cultivation** | |
|---|---|---|---|---|
| | **Labour (d/ha/yr)** | **Inputs ($/ha)** | **Labour (d/ha/yr)** | **Inputs ($/ha)** |
| Year 0 | 36.5 | $353 | 27 | $623 |
| Year 1-20 | 31.5 | $353 | 22 | $623 |

Maize is not cultivated every year in the alley cropping system, and therefore (unlike the monoculture system) high fertiliser inputs are not needed to sustain constant annual yields over the 20-year period. When growing maize as a monoculture MIDA (2019b) recommends applying 272 kg of both complex NPK fertiliser and urea per hectare. For maize cultivation under alley cropping, we apply this amount of NPK fertiliser but no urea. Furthermore, to conform with the requirements of the Forest Stewardship Council, no pesticides are applied in the alley cropping system.

We reduced the labour days and input costs needed for sowing, fertilising and harvesting maize in the alley cropping system by a factor of 0.83, to account for the reduced area on which these activities occur (compared to the monoculture system). However, we also included two additional labour days per hectare for harvesting maize in the agroforestry system, to reflect the extra time and care needed to protect trees from mechanical damage (Paul et al. 2017). When increased shading reduces expected maize yields by 50%, we apply half the fertiliser and reduce harvest, threshing and transport costs by 50%.

### *1.2. Pasture and timber-based systems*

Table S2 outlines the establishment and ongoing management costs of pasture, teak plantation and the two agroforestry systems.

**Table S2** Labour and inputs costs over the 20-year period for teak plantation, alley cropping, silvopasture and pasture. Cost follow Paul (2014) and Paul et al. (2015) unless noted otherwise.

| | | **Teak plantation** | | **Alley cropping** | | **Silvopasture** | | **Pasture** | | |
|---|---|---|---|---|---|---|---|---|---|---|
| | **Year** | **Labour (d/ha/yr)** | **Inputs ($/ha)** | **Labour (d/ha/yr)** | **Inputs ($/ha)** | **Labour (d/ha/yr)** | **Inputs ($/ha)** | **Labour (d/ha/yr)** | **Inputs ($/ha)** | **Source/comment** |
| Field preparation | 0 | 16 | Herbicide: $18 | 16 | Herbicide: $18 | 16 | Herbicide: $18 | 11 | Herbicide: $18 | Manual and chemical removal of secondary vegetation and weeds. Includes 5 extra labour days for timber-based systems to account for higher planning complexity (Paul et al. 2017). |
| Tree establishment | 0 | 33 | Trees: $555<br>Fertiliser: $33 | 16.5 | Trees: $278<br>Fertiliser: $17 | 8.5 | Trees: $100<br>Fertiliser: $6<br>Tree guards: $150 | NA | NA | Tree seedlings cost $0.50 each, 50 g NPK fertilizer applied to each tree. |
| Replanting trees | 0 | 9 | Trees: $166<br>Fertiliser: $10 | 5 | Trees: $83<br>Fertiliser: $5 | 0.5 | Trees: $7 | NA | NA | Assumed 30% tree mortality in the first year for teak and 7% for cedar, according to experience in the trial in Tortí (Paul 2014; Paul et al. 2015). |
| Pasture establishment and fencing | 0 | NA | NA | NA | NA | 8.2 | Pasture seeds: $84<br>Fencing material: $226 | 8.2 | Pasture seeds: $84<br>Fencing material: $226 | Fencing costs are taken from MIDA 2016 and assume a mean paddock size of 5 ha. |
| Cattle costs | 0-19 | NA | NA | NA | NA | - | $807-$611 | - | $807 | Cost of purchasing cattle and vaccinations: costs decline in the silvopastoral system as the stocking rate falls with increased shading. |
| Spot-ring weeding | 0<br>1<br>2 | 22<br>5.5<br>- | -<br>-<br>- | 11<br>2.8<br>- | -<br>-<br>- | 4<br>4<br>4 | -<br>-<br>- | NA | NA | Manual removal of all vegetation within 1m of trees using a machete. |
| Maize cultivation | 0<br>1-2<br>6 & 11 | NA | NA | 14.5<br>18.5<br>14 | $265<br>$265<br>$132 | NA | NA | NA | NA | Less fertiliser and pesticide inputs compared to maize monoculture. In Yr 0 site already prepared in the course of tree establishment. In years 6 and 11, less fertiliser is applied and lower yields reduce harvesting, threshing and transport costs. |
| Manual weeding | 1<br>2<br>3<br>4+ | 12<br>20<br>12<br>4 | -<br>-<br>-<br>- | 2.8<br>-<br>6<br>2 (0 if maize cultivated) | -<br>-<br>-<br>- | -<br>-<br>-<br>- | -<br>-<br>-<br>- | NA | NA | Less intensive manual weeding. In the alley cropping system, this type of weeding is carried out during maize cultivation. |

| | Year | Teak plantation Labour (d/ha/yr) | Teak plantation Inputs ($/ha) | Alley cropping Labour (d/ha/yr) | Alley cropping Inputs ($/ha) | Silvopasture Labour (d/ha/yr) | Silvopasture Inputs ($/ha) | Pasture Labour (d/ha/yr) | Pasture Inputs ($/ha) | Source/comment |
|---|---|---|---|---|---|---|---|---|---|---|
| Pest control (*H. grandella*) | 1-3 | NA | NA | NA | NA | 6.5 | - | NA | NA | Monitoring trees four times a year and removing infected branches. |
| Pruning | 1 | 12 | $10 | 6 | $10 | - | - | | | Inputs refer to base costs, which cover tools and materials that may not be available on farms. |
| | 2 | 12 | $10 | 6 | $10 | - | - | | | |
| | 3 | - | - | 6 | $10 | - | - | | | |
| | 4 | 12 | $10 | - | - | 2 | $10 | NA | NA | |
| | 5 | - | - | 3 | $10 | 2 | $10 | | | |
| | 6 | - | - | - | - | 2 | $10 | | | |
| | 7 | - | - | - | - | 2 | $10 | | | |
| Pasture management | 2-20 | NA | NA | NA | NA | 2 | Seeds: $24 Herbicide: $4.5 | 2 | Seeds: $24 Herbicide: $4.5 | Spot-spraying weeds and replanting patches of pasture that have died. |
| Thinning | 4 | 33 | Base cost: $127 Machines costs: $65 | - | - | - | - | | | Thinning and harvesting costs based on three dbh size classes: 0-14 cm (first thinning), 15-20 cm (second thinning) and >20 cm (final harvest). Base costs cover tools and equipment and preparing the site and maintaining infrastructure. |
| | 5 | - | - | 14 | Base cost: $127 Machine costs: $27 | - | - | NA | NA | |
| | 10 | 36 | Base cost: $127 Machine costs: $105 | 23 | Base cost: $127 Machine costs: $66 | - | - | | | |
| Final harvest | 20 | 53 | Base cost: $127 Machine costs: $150 | 33 | Base cost: $127 Machine costs: $95 | 48 | Base cost: $127 Machine costs $136 | NA | NA | |
| Ongoing management | 1-20 | - | Fixed costs: $60 | - | Fixed costs: $60 | 6 | Fixed costs: $60 Transport: $40 | 6 | Transport: $40 | The timber systems incur an annual fixed cost of $60 per hectare to account for the higher complexity of these systems compared to pure agriculture, and therefore the need for more technical assistance and monitoring. In the cattle systems labour includes fence maintenance, monitoring cows, applying vaccinations, buying and selling cows (Paul et al. 2015). |

## 2. Modelling tree growth and canopy shading

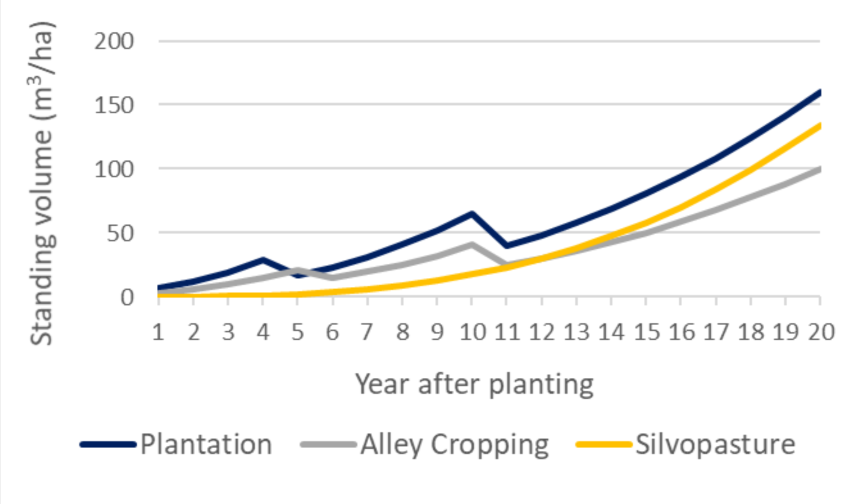


**Fig. S1** Projected standing timber volume ($m^3$/ha) of each timber system over the 20-year rotation.

**Table S3** Projected tree dimensions at age 20.

| | Height (m) | Dbh (cm) | Crown radius (m) | Comment |
|---|---|---|---|---|
| Teak | 22.6 | 29.9 | 4.5 | In line with growth measurements from a teak plantation in a comparable site in western Panama (Las Lajas, Chiriquí; Paul et al. 2015), as well as biophysical modelling for the study area using WaNuLCAS (Paul et al. 2017). |
| Cedar | 24 | 28 | 2.6 | Cedar growth rate within range reported for plantations in Latin America (Cintron 1990) and Costa Rica (Bellow and Nair 2003). Compact crown plausible given the prolonged pruning regime from years four to seven. |

**Table S4** Categories for yield reductions of maize in the alley cropping system based on canopy shading and tree heights – adopted from Paul et al. (2015).

| Percent canopy shading | Tree height (m) | Yield reduction factor |
|---|---|---|
| ≤ 15 % | ≤ 3 | 1.00 |
| 16-35 % | ≤ 3 | 0.75 |
| 16-35 % | > 3 | 0.50 |
| 36-55 % | ≤ 3 | 0.75 |
| 36-55 % | 3.1 - 6 | 0.50 |
| 36-55 % | > 6 | 0 |
| > 55 % | ≤ 6 | 0.50 |
| > 55 % | > 6 | 0 |

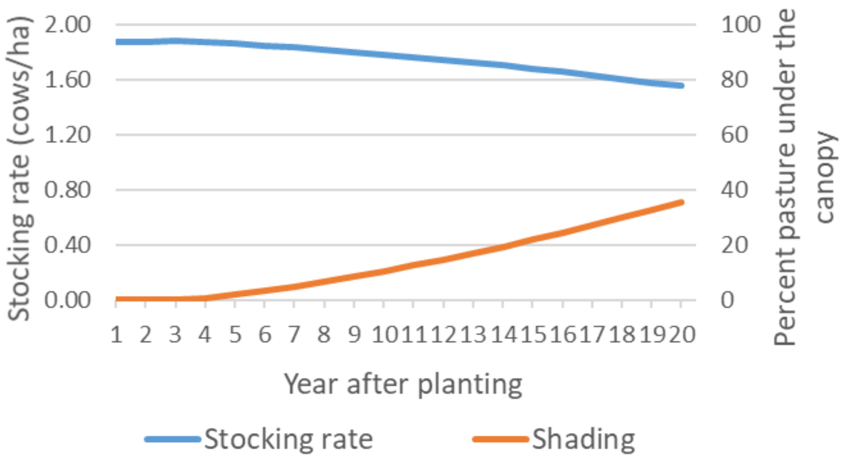


**Fig. S2** Increase in shading and subsequent decrease in stocking rate of the silvopastoral system over the 20-year rotation.

## 3. Revenues (expected yields and prices)

### *3.1. Revenue from crops and cattle*

Expected yields and producer prices for each agricultural crop and cattle are shown in Table S5. Rice and maize yields are compiled at the national level for traditional (non-mechanised) planting systems with use of pesticides and fertilisers. For maize grown in the alley cropping system, we reduce the initial expected hectare yield by a factor of 0.83, to account for the smaller area covered by the crop.

**Table S5** Expected yields and prices for each agricultural crop (years 0-20) and pasture (years 1-20), based on information from MIDA and local experts (Reyes Cáceres 2018).

| Crop | Expected yield (ton/ha/yr) | Price ($/ton) | Expected revenue ($/ha/yr) | Source |
|---|---|---|---|---|
| Rice | 2.7 | 539 | 1466 | MIDA 2019a |
| Maize | 3.4 | 442 | 1518 | MIDA 2019b |
| Pasture (meat liveweight) | 0.9 | 1550 | 1407 | Reyes Cáceres 2018 |

In the cattle systems young cows are bought with an initial weight of 250 kg and fattened to 454 kg by the end of the 12-month ceba period. Based on knowledge of local farmers and agricultural experts we selected a stocking rate of 2 cows per hectare for conventional pasture. Beef prices are taken from the local cattle market. The initial stocking rate of the silvopastoral system is reduced by a factor of 0.95 to account for the reduced pasture area: the stocking rate then declines linearly with the canopy growth of cedar trees (see Figure S2 and Equation 1 in the main text).

### *3.2. Timber revenues*

Following Griess and Knoke (2011) and Paul et al. (2017), only 64% of the standing wood volume was considered to be marketable timber: this accounts for 20% harvests losses and assumes that 80% of the harvested volume is stem wood. Prices for teak and cedar logs were obtained from the National Forest Office (ONF) in Costa Rica (ONF 2019) – see Table S6.  Prices for the very small logs (<15 cm dbh) are taken from Paul et al. (2017). We applied a 0.5 reduction factor to the cedar price (ONF 2019), because timber quality is likely to be much lower in a silvopastoral system compared to plantation (Love et al. 2009).

**Table S6** Expected timber yields and prices for plantations, alley cropping and silvopasture (ONF 2019; Paul et al. 2017).

| | Year | Dbh (cm) | Expected yield ($m^3$/ha) | Expected price (\$/$m^3$) | Expected revenue (\$/ha) |
|---|---|---|---|---|---|
| Plantation | | | | | |
| First thinning | 4 | 9.1 | 11.2 | 50 | 560 |
| Second thinning | 10 | 16.9 | 20.7 | 160 | 3323 |
| Final harvest | 20 | 29.9 | 102.5 | 234 | 24044 |
| Alley cropping | | | | | |
| First thinning | 5 | 10.4 | 6.7 | 50 | 336 |
| Second thinning | 10 | 16.9 | 12.9 | 160 | 2077 |
| Final harvest | 20 | 29.9 | 64.1 | 234 | 15028 |
| Silvopasture | | | | | |
| Final harvest | 20 | 24.0 | 86.0 | 123 | 10533 |

### *3.3. Net cash flow*

Using the expected costs and revenues for each land-use, *l*, we determined the net cash flow (NCF) for each land-use for each year, *t*, of the 20-year period (Table S8):

$$NCF_{l,t} = Expected\ Revenues_{l,t} - Expected\ Cost_{l,t} \qquad (S1)$$

**Table S7** Nominal net cash flows (\$/ha) for each land-use over the 20-year period.

| | **Year, *t*** | | | | | | | | | | |
|---|---|---|---|---|---|---|---|---|---|---|---|
| **Land-use** | **0** | **1** | **2** | **3** | **4** | **5** | **6** | **7** | **8** | **9** | **...** |
| Rice | 517 | 603 | 603 | 603 | 603 | 603 | 603 | 603 | 603 | 603 | |
| Maize | 444 | 531 | 531 | 531 | 531 | 531 | 531 | 531 | 531 | 531 | |
| Pasture | -1435 | 456 | 393 | 393 | 393 | 393 | 393 | 393 | 393 | 393 | |
| Plantation | -2185 | -581 | -485 | -199 | -423 | -129 | -129 | -129 | -129 | -129 | |
| Alley cropping | -817 | 130 | 178 | -242 | -95 | -209 | -31 | -95 | -95 | -95 | |
| Silvopasture | -1970 | 177 | 114 | 183 | 248 | 244 | 240 | 236 | 278 | 272 | |
| Forest | 0 | 0 | 0 | 0 | 0 | 0 | 0 | 0 | 0 | 0 | |

| | **Year, *t* (continued)** | | | | | | | | | | |
|---|---|---|---|---|---|---|---|---|---|---|---|
| **Land-use** | **10** | **11** | **12** | **13** | **14** | **15** | **16** | **17** | **18** | **19** | **20** |
| Rice | 603 | 603 | 603 | 603 | 603 | 603 | 603 | 603 | 603 | 603 | 603 |
| Maize | 531 | 531 | 531 | 531 | 531 | 531 | 531 | 531 | 531 | 531 | 531 |
| Pasture | 393 | 393 | 393 | 393 | 393 | 393 | 393 | 393 | 393 | 393 | 1168 |
| Plantation | 2336 | -129 | -129 | -129 | -129 | -129 | -129 | -129 | -129 | -129 | 22710 |
| Alley cropping | 1398 | -31 | -95 | -95 | -95 | -95 | -95 | -95 | -95 | -95 | 14132 |
| Silvopasture | 267 | 261 | 256 | 249 | 243 | 236 | 229 | 222 | 214 | 206 | 10234 |
| Forest | 0 | 0 | 0 | 0 | 0 | 0 | 0 | 0 | 0 | 0 | 0 |

## 4. Adding elements of uncertainty

**Table S8** Producer Price index (2004-2006 = 100) for crops and beef in Panama, obtained from the FAO Corporate Statistical Database (FAOSTAT). CV = coefficient of variation.

| Year, *h* | Rice ($/ton) | Maize ($/ton) | Beef ($/kg) |
|---|---|---|---|
| 1997 | 101 | 105 | 109 |
| 1998 | 104 | 108 | 109 |
| 1999 | 100 | 109 | 121 |
| 2000 | 105 | 109 | 115 |
| 2001 | 95 | 103 | 115 |
| 2002 | 98 | 99 | 119 |
| 2003 | 97 | 103 | 99 |
| 2004 | 100 | 103 | 100 |
| 2005 | 97 | 98 | 100 |
| 2006 | 103 | 98 | 100 |
| 2007 | 102 | 113 | 102 |
| 2008 | 148 | 153 | 107 |
| 2009 | 155 | 168 | 113 |
| 2010 | 163 | 161 | 123 |
| 2011 | 161 | 207 | 128 |
| 2012 | 206 | 251 | 141 |
| 2013 | 212 | 241 | 144 |
| 2014 | 190 | 208 | 145 |
| 2015 | 204 | 234 | 144 |
| 2016 | 208 | 217 | 141 |
| CV | 0.33 | 0.38 | 0.41 |

**Table S9** Mean crop and beef yields in Panama from 1997 to 2016, obtained from FAOSTAT. CV = coefficient of variation.

| Year, *h* | Rice (ton/ha) | Maize (ton/ha) | Beef (kg/animal) |
|---|---|---|---|
| 1997 | 2.20 | 1.25 | 223 |
| 1998 | 2.63 | 1.62 | 223 |
| 1999 | 2.88 | 1.56 | 223 |
| 2000 | 2.66 | 1.51 | 223 |
| 2001 | 3.17 | 1.36 | 223 |
| 2002 | 3.08 | 1.51 | 223 |
| 2003 | 3.27 | 1.54 | 223 |
| 2004 | 2.46 | 1.52 | 223 |
| 2005 | 3.02 | 1.49 | 223 |
| 2006 | 2.64 | 1.76 | 223 |
| 2007 | 2.95 | 1.74 | 217 |
| 2008 | 3.12 | 1.69 | 217 |
| 2009 | 2.98 | 1.78 | 217 |
| 2010 | 2.61 | 1.40 | 217 |
| 2011 | 2.95 | 1.86 | 217 |
| 2012 | 2.88 | 2.02 | 217 |
| 2013 | 3.09 | 2.36 | 217 |
| 2014 | 3.26 | 2.56 | 217 |
| 2015 | 3.05 | 1.56 | 217 |
| 2016 | 3.52 | 2.17 | 217 |
| CV | 0.11 | 0.20 | 0.01 |

We account for yield and price variability through Monte Carlo simulations, in which expected prices and yields are adjusted based on historical data. For each simulation run, *a*, the bootstrapping process (random sampling with replacement) selects a year, *h*, from the historic dataset for each year, *t*, of the land-use model. The randomly chosen year is used to adjust the expected price (Equation S2) and expected yield (Equation S3) of each commodity, c, for that year of the land-use model to represent market and yield fluctuations respectively. The quotient of a given commodity price or yield for the randomly selected year, *h*, and the average price or yield for that commodity (2007-2016) is multiplied by expected price or yield in the model:

$$Price_{t,c,a} = \frac{Price_{h,c}}{\frac{1}{H}\sum_{h=1}^{H} Price_{h,c}} \times Expected\ price_{c,t} \quad \text{for c = rice, maize and beef} \quad \text{(S2)}$$

$$Yield_{t,c,a} = \frac{Yield_{h,c}}{\frac{1}{H}\sum_{h=1}^{H} Yield_{h,c}} \times Expected\ yield_{c,t} \quad \text{for c = rice, maize and beef} \quad \text{(S3)}$$

Reliable data on historic timber yields and prices were not available for Panama. Therefore, following Paul et al. (2017) and Castro et al. (2015) we assume a 10% coefficient of variation for timber yields. For prices we assume a 19% coefficient of variation for teak, and 8% coefficient of variation for cedar, based on the variation of timber prices in Costa Rica between 2007 and 2020 (ONF 2020). Within the land-use model, timber price and yields were adjusted accordingly for each simulation run.

Simulating variation in yield and prices drives variation in three of the socio-economic indicators: NPV, payback periods and food production. To simulate variation in labour demand and investment costs we randomly selected values from the normal distribution, assuming a 10% coefficient of variation in investment costs and yearly labour demand.

By repeating the Monte Carlo simulation 10,000 times we generated a frequency distribution for each indicator. From these 10,000 simulation runs we then calculated the mean (predicted) value $\hat{y}_{i,l}$ and standard deviation $SD_{i,l}$ of each indicator for each land-use:

$$\hat{y}_{i,l} = \frac{1}{10000} \sum_{a=1}^{a} y_{i,l,a} \quad \text{(S4)}$$

$$SD_{i,l} = \sqrt{\frac{\sum (y_{i,l,a} - \hat{y}_{i,l})^2}{9999}} \quad \text{(S5)}$$

## 5. Computing energy production

**Table S10** Energy content and technical conversion factors for each food commodity.

| | Energy content (kcal per 100g) | Yield conversion factor | Source/comment |
|---|---|---|---|
| Rice | 370 | 0.63 | 3.8% yield losses within supply chain until food consumption in Panama, with an extraction coefficient of 65% (FAO 2019). |
| Maize | 365 | 0.79 | 1.5% yield losses within supply chain until food consumption in Panama (FAO 2019), shelling fraction of 80% is typical for Central America (Bolaños 1995). |
| Beef | 291 | 0.37 | Carcass weight 52% of live weight, 71% of which is boneless meat (FAO 2019). |

## 6. Formulation of multi-criteria optimisation model

The starting point for the multi-criteria optimisation model are the predicted values $\hat{y}_{i,l}$, which form our estimate of the ability of each land-use, *l*, to achieve each indicator, *i*. However, recognising the uncertainty associated with these estimates, the model also considers potential deviations based on the standard deviation, $SD_{i,l}$. To do so we compute uncertainty adjusted values, $y_{i,l,u}$ that span from a best- to worst-case estimate. For our best-case we take the mean score, while for the worst-case estimate we add or subtract a multiple, *m*, of the SD, depending on the direction of the indicator:

$$y_{i,l,u} = \begin{cases} \hat{y}_{i,l} & \text{for best case} \\ \hat{y}_{i,l,} - m \cdot SD_{i,l,} & \text{for worst case, if more is considered better} \\ \hat{y}_{i,l,} + m \cdot SD_{i,l,} & \text{for worst case, if less is considered better} \end{cases} \qquad \text{(S6)}$$

The model generates all possible combinations of best- and worst-case estimates across the considered land-uses in discrete uncertainty scenarios. This results in $2^L$ scenarios per indicator, where *L* is the number of land-uses considered in the optimisation. These uncertainty scenarios describe the surface (i.e. provide the corner points) of multi-dimensional boxes that represent our uncertainty spaces $U_i$ for each indicator. The optimisation considers all corner points simultaneously. Therefore the optimal land allocation offers a feasible solution for all input values contained within the uncertainty spaces (Knoke et al. 2020). In our study the optimisation considers 640 uncertainty scenarios ($2^7$ scenarios × 5 indicators).

Within each uncertainty scenario the model computes the performance of a hypothetical farm portfolio for achieving a given objective. The hypothetical farm portfolio comprises various shares, $a_l$, of each land-use. We compute the farm-level performance, $Y_{i,u}$, of this portfolio as the sum of the uncertainty adjusted values $y_{i,l,u}$ (i.e. best and worst case estimates) within a given scenario, *u*, weighted by the area share, $a_l$, of each land-use in the hypothetical portfolio:

$$Y_{i,u} = \sum_l y_{i,l,u} a_l \qquad \text{(S7)}$$

The unit of this farm-level performance depends on the indicator (e.g. for NPV it is measured in $/ha, for food production Mcal/ha/yr). Therefore, to compare performance across different indicators, we normalise $Y_{i,u}$ between 0 and 100%. To do so we set the best performing uncertainty adjusted value within each uncertainty scenario as our reference point, i.e. the 100% or target level. For "more is better" indicators the reference point is the highest uncertainty adjusted value within an uncertainty scenario, $y^*_{i,u(m=3)} = \max_l\{y_{i,l,u(m=3)}\}$, while for less is better indicators it is the lowest, $y_{i,u(m=3)*} = \min_l\{y_{i,l,u(m=3)}\}$. To ensure robust results, reference points are computed across all uncertainty levels using m = 3, denoted as $u(m = 3)$. For each uncertainty scenario we divide the difference between the reference point and farm-level performance by the difference between the highest and lowest uncertainty-adjusted values ($\Delta_{i,u(m=3)}$), to produce a normalised distance $D_{i,u}$ to the 100% level:

$$D_{i,u} = \begin{cases} \frac{y^*_{i,u(m=3)} - Y_{i,u}}{\Delta_{i,u(m=3)}} \cdot 100 \text{ if more is better} \\ \frac{Y_{i,u} - y_{i,u(m=3)*}}{\Delta_{i,u(m=3)}} \cdot 100 \text{ if less is better} \end{cases} \qquad \text{(S8)}$$

$$\Delta_{i,u(m=3)} = y^*_{i,u(m=3)} - y_{i,u(m=3)*} \qquad \text{(S9)}$$

The variable $D_{i,u}$ measures the shortfall between farm-level performance for a given indicator and the target (best possible) level, which can be interpreted as underperformance. We define $\beta$ as the maximum $D_{i,u}$ across all uncertainty scenarios:

$$\beta = \max_{i,u}\{D_{i,u}\} \qquad \text{(S10)}$$

The variable $\beta$ serves as our objective function, which we want to minimise. To solve the allocation problem we set area shares, $a_l$, allocated to each land-use as the decision variables. Equations S11 to S14 formulate the optimisation problem.

$$\text{Minimise } \beta \qquad \text{(S11)}$$

subject to:

$$\beta \geq D_{i,u} \quad \forall_i \in \mathrm{I},\ \forall_u \in U_i \qquad \text{(S12)}$$

$$\textstyle\sum_l a_l = 1 \qquad \text{(S13)}$$

$$a_l \geq 0 \qquad \text{(S14)}$$

By minimising $\beta$ we aim to minimise the worst underperformance (highest $D_{i,u}$) across all indicators. Inequation 12 is needed to linearise the objective function; the inequation summarises individual constraints (representing $2^L$ uncertainty scenarios per indicator) as $\beta$ on the left side and the level of underperformance on the right side. This formulation of the problem can be solved exactly by the Simplex algorithm.

## 7. Scenario analysis

### *7.1. Prioritising individual objectives*

In the scenario *Prioritising individual objectives* we test the effect of weighing individual objectives above the others. We derived weights, $w_i$, by assuming an individual indicator is twice as important as the others (Table S11). For each uncertainty scenario, *u*, and indicator, *i*, we multiplied the distance between the achieved and target indicator level, $D_{i,u}$, by the weight, $w_i$, of each indicator. The model then determined the land-use composition that minimised the maximum of these weighted distances.

$$\text{Minimise } \beta\text{, where } \beta = \max_{i,u}\{D_{i,u} \cdot w_i\} \qquad \text{(S15)}$$

**Table S11** Relative weighting of indicators when net present value (NPV) is given twice as much importance as the other objectives.

| Indicator (*i*) | Relative importance | Weight ($w_i$) |
|---|---|---|
| NPV | 2 | 0.33 |
| Payback period | 1 | 0.17 |
| Food production | 1 | 0.17 |
| Labour demand | 1 | 0.17 |
| Investment costs | 1 | 0.17 |
| Total | 6 | 1.00 |

### *7.2. Farmer preferences*

In the *Farmer preferences* scenario we include the general land-use preferences obtained through farmer interviews (Gosling et al. 2020, see Table S12) as an additional indicator in the multi-criteria

optimisation model. These stated land-use preferences may serve as a proxy for more intangible or cultural values that are not easily captured by the other socio-economic indicators (Knoke et al. 2014; Temesgen and Wu 2018). Therefore, including them as an additional (equally weighted) indicator in the multi-criteria model can help to test how farmers' cultural values may influence the optimal land-use composition.

**Table S12** Farmers' general land-use preference scores obtained in farmer interviews. Scores represent the number of times a land-use was selected as the best or second best land-use option, showing the standard error of the estimate. Data taken from Gosling et al. 2020.

| Crop | Preference score | |
|---|---|---|
| Rice | 15.0 | ± 3.44 |
| Maize | 15.0 | ± 3.44 |
| Pasture | 21.0 | ± 3.85 |
| Teak plantation | 0.0 | ± 0.00 |
| Alley Cropping | 11.0 | ± 3.05 |
| Silvopasture | 23.0 | ± 3.94 |
| Forest | 1.0 | ± 0.99 |

## 8. Supplementary results

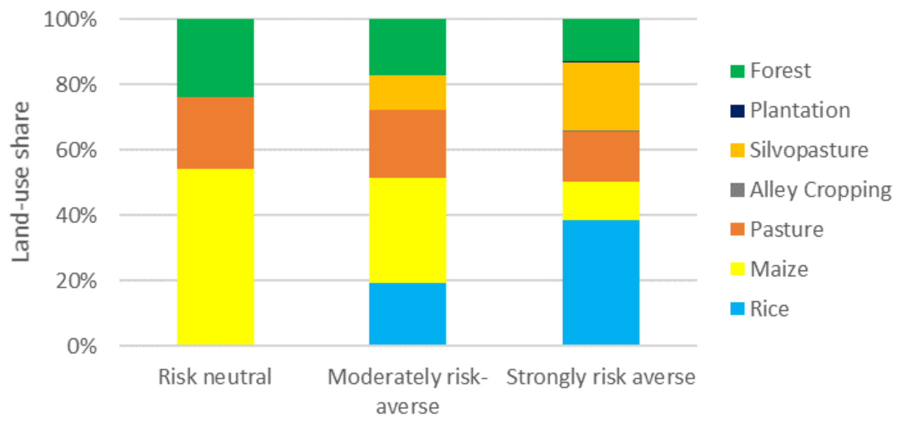


**Fig. S3** Composition of the optimised farm portfolio (share of land area allocated to each land-use) for three levels of risk aversion: risk neutral (m = 0), moderately risk-averse (m = 1.5), and strongly risk-averse (m = 3.0), when farmers' land-use preferences (see Table S12) are included as an additional (equally weighted) indicator in the multi-criteria model.

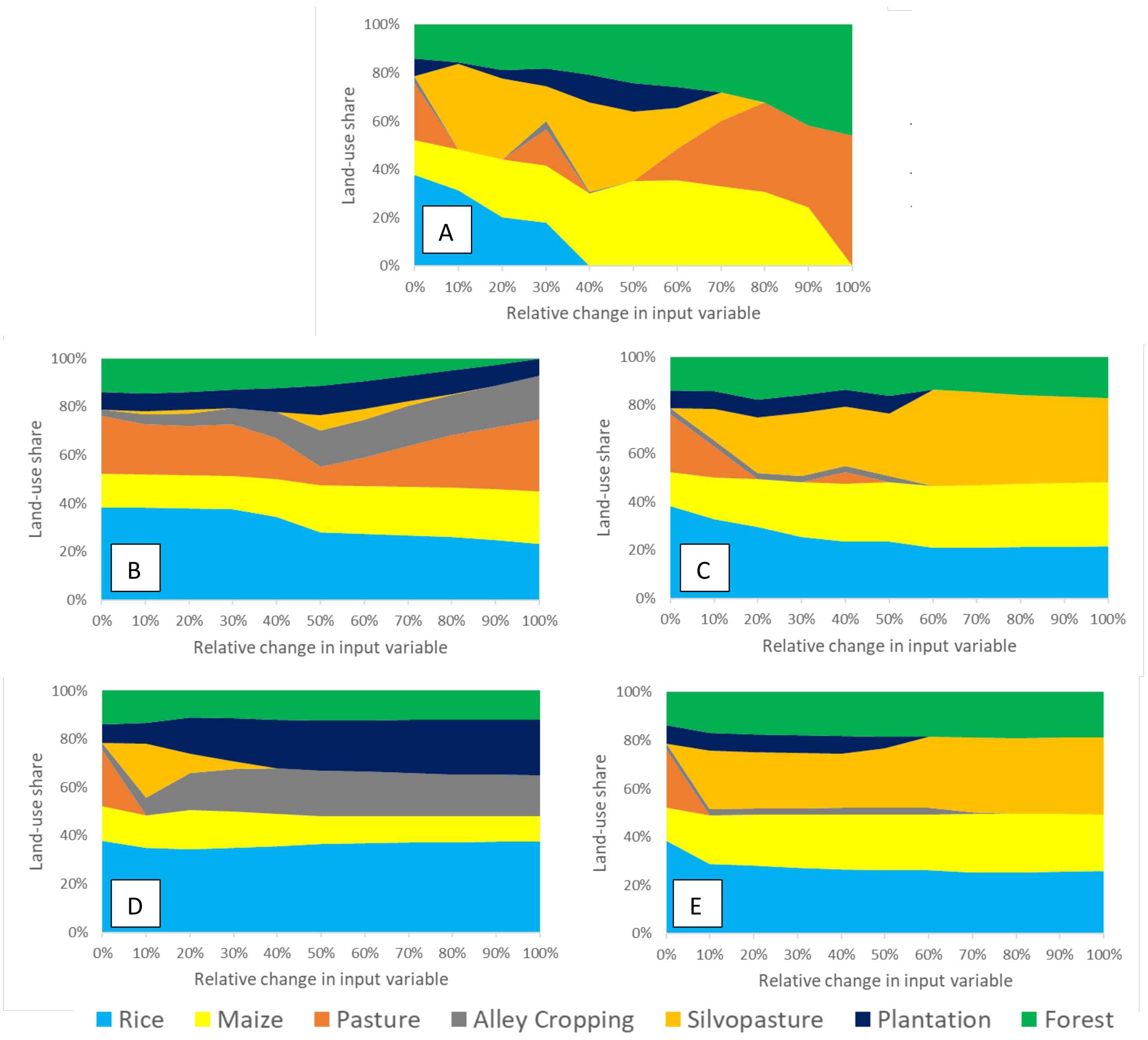


**Fig. S4** Composition of the ideal farm (share of land area allocated to each land-use) for a strongly risk-averse farmer (m = 3.0), under different input variable scenarios: A) declining yields of annual crops (maize and rice), B) decreasing investment costs of alley cropping, C) decreasing investment costs of silvopasture, D) increasing teak prices and E) increasing cedar prices.

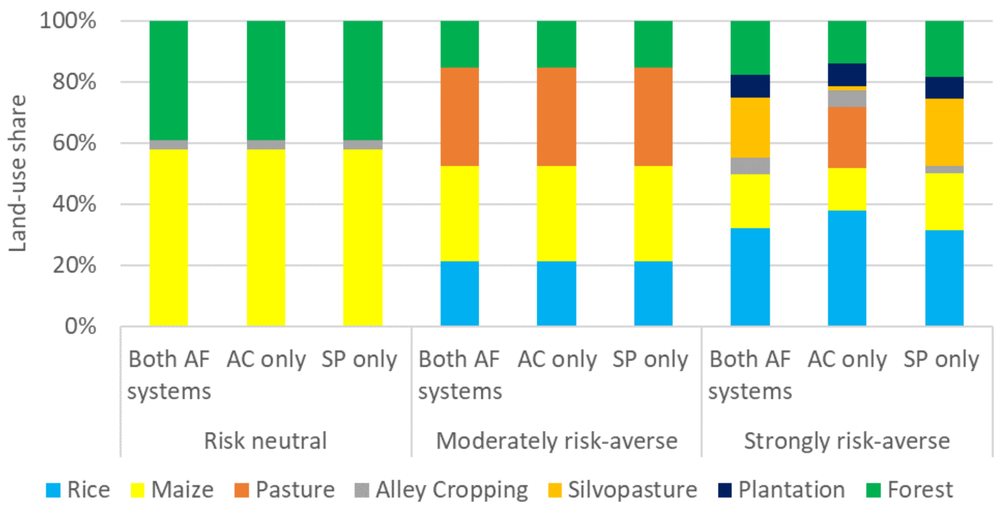


**Fig. S5** Composition of the ideal farm (share of land area allocated to each land-use) when farmers are provided with a) free trees and tree guards for both agroforestry systems b) for alley cropping only or c) for silvopasture only. Results are given for three levels of risk aversion: a risk neutral farmer (m = 0, leftmost three columns), a moderately risk-averse farmer (m = 1.5, middle three columns) and a strongly risk-averse farmer (m = 3.0, rightmost three columns).

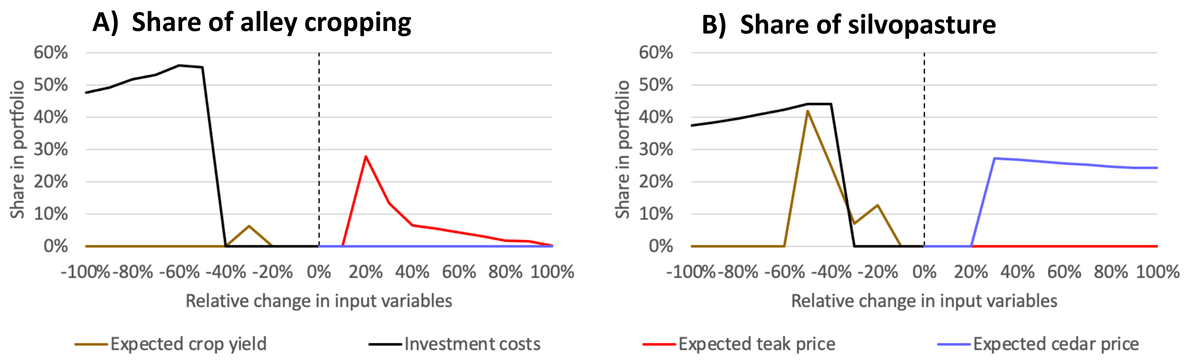


**Fig. S6:** Share of a) alley cropping and b) silvopasture selected in the optimal land-use portfolio when changing the assumptions and coefficients of the land-use model. Input variables of the land-use model are progressively increased or decreased under three scenarios: changes to expected crop yields relate to the *Lower crop yields* scenario, changes in investment costs to *Agroforestry subsidy* and changes in teak and cedar price to *Higher timber prices*. These scenarios are described in Table 5 in the main text. Optimisation carried out from the perspective of a moderately risk-averse decision-maker (m = 1.5).

## References

Bellow J, Nair PKR (2003) Comparing common methods for assessing understory light availability in shaded-perennial agroforestry systems. Agricultural and Forest Meteorology 114:197–211. https://doi.org/10.1016/S0168-1923(02)00173-9

Bolaños J (1995) Physiological bases for yield differences in selected maize cultivars from Central America. Field Crops Research 42:69–80. https://doi.org/10.1016/0378-4290(95)00022-I

Castro LM, Calvas B, Knoke T (2015) Ecuadorian banana farms should consider organic banana with low price risks in their land-use portfolios. PLoS ONE 10:e0120384. https://doi.org/10.1371/journal.pone.0120384

Cintron BB (1990) Cedrela oderata, L. In: Burns RM, Honkala BH (eds) Silvics of North America. U.S. Dept. of Agriculture Forest Service, Washington, 250-257

FAO (2019) Technical conversion factors for agricultural commodities. www.fao.org/economic/the-statistics-division-ess/methodology/methodology-systems/technical-conversion-factors-for-agricultural-commodities/en. Accessed 14 August 2019

Gosling E, Reith E, Knoke T, Paul C (2020) A goal programming approach to evaluate agroforestry systems in Eastern Panama. J Environ Manage 261:110248. https://doi.org/10.1016/j.jenvman.2020.110248

Griess VC, Knoke T (2011) Can native tree species plantations in Panama compete with teak plantations?: An economic estimation. New Forests 41:13–39. https://doi.org/10.1007/s11056-010-9207-y

Knoke T, Bendix J, Pohle P, Hamer U, Hildebrandt P, Roos K, Gerique A, Sandoval ML, Breuer L, Tischer A, Silva B, Calvas B, Aguirre N, Castro LM, Windhorst D, Weber M, Stimm B, Günter S, Palomeque X, Mora J, Mosandl R, Beck E (2014) Afforestation or intense pasturing improve the ecological and economic value of abandoned tropical farmlands. Nat Commun 5:5612. https://doi.org/10.1038/ncomms6612

Knoke T, Paul C, Rammig A, Gosling E, Hildebrandt P, Härtl F, Peters T, Richter M, Diertl K-H, Castro LM, Calvas B, Ochoa S, Valle-Carrión LA, Hamer U, Tischer A, Potthast K, Windhorst D, Homeier J, Wilcke W, Velescu A, Gerique A, Pohle P, Adams J, Breuer L, Mosandl R, Beck E, Weber M, Stimm B, Silva B, Verburg PH, Bendix J (2020) Accounting for multiple ecosystem services in a simulation of land-use decisions: Does it reduce tropical deforestation? Global Change Biology. https://doi.org/10.1111/gcb.15003

Love BE, Bork EW, Spaner D (2009) Tree seedling establishment in living fences: A low-cost agroforestry management practice for the tropics. Agroforest Syst 77:1–8. https://doi.org/10.1007/s10457-009-9244-8

MIDA (2016) Direccion de ganaderia: Costo de 1 kilometro de cerca perimetral. November 2016. Ministerio de Desarrollo Agropecuario (MIDA), Panama.

MIDA (2019a) Direccion de agricultura: Costo de produccion normativo de una hectarea de maiz. Ministerio de Desarrollo Agropecuario (MIDA). https://www.mida.gob.pa/direcciones/direcciones_nacionales /direcci-n-de-agricultura/costos-de-producci-n-2019.html. Accessed 12 August 2019

MIDA (2019b) Direccion de agricultura: Costo de produccion normativo de una hectarea de arroz. Ministerio de Desarrollo Agropecuario (MIDA). https://www.mida.gob.pa/direcciones/direcciones_nacionales /direcci-n-de-agricultura/costos-de-producci-n-2019.html. Accessed 12 August 2019

ONF (2019) Precios de la madera: Para las especies mas comercializadas. Primer semestre del 2019. Oficina Nacional Forestal de Costa Rica (ONF). https://onfcr.org/informe-de-precios-de-madera/. Accessed 14 August 2019

ONF (2020) Precios de la madera: Para las especies mas comercializadas. Primer semestre del 2020. Oficina Nacional Forestal de Costa Rica (ONF). https://onfcr.org/informe-de-precios-de-madera/. Accessed 23 July 2020

Paul C (2014) Timber-based agrisilvicultural systems to facilitate reforestation in Panama: A silvicultural and economic evaluation. Dissertation, Technical University of Munich

Paul C, Griess VC, Havardi-Burger N, Weber M (2015) Timber-based agrisilviculture improves financial viability of hardwood plantations: A case study from Panama. Agroforest Syst 89:217–235. https://doi.org/10.1007/s10457-014-9755-9

Paul C, Weber M, Knoke T (2017) Agroforestry versus farm mosaic systems - Comparing land-use efficiency, economic returns and risks under climate change effects. Science of the Total Environment 587-588:22–35. https://doi.org/10.1016/j.scitotenv.2017.02.037

Reyes Cáceres A (2018) Assessing the economic potential of agroforestry systems in Tortí, Panama. Master’s thesis, Technical University of Munich

Temesgen H, Wu W (2018) Farmers’ value assessment of sociocultural and ecological ecosystem services in agricultural landscapes. Sustainability 10:703. https://doi.org/10.3390/su10030703
